# Transcriptional reprogramming of nucleotide metabolism in response to altered pyrimidine availability in Arabidopsis seedlings

**DOI:** 10.3389/fpls.2023.1273235

**Published:** 2023-11-02

**Authors:** Robert D. Slocum, Carolina Mejia Peña, Zhongchi Liu

**Affiliations:** ^1^ Department of Biological Sciences, Goucher College, Towson, MD, United States; ^2^ Department of Molecular Biology, Cell Biology, and Biochemistry, Brown University, Providence, RI, United States; ^3^ Department of Cell Biology and Molecular Genetics, University of Maryland, College Park, MD, United States

**Keywords:** nucleotide metabolism, pyrimidine, purine, *Arabidopsis*, transcriptome

## Abstract

In Arabidopsis seedlings, inhibition of aspartate transcarbamoylase (ATC) and *de novo* pyrimidine synthesis resulted in pyrimidine starvation and developmental arrest a few days after germination. Synthesis of pyrimidine nucleotides by salvaging of exogenous uridine (Urd) restored normal seedling growth and development. We used this experimental system and transcriptional profiling to investigate genome-wide responses to changes in pyrimidine availability. Gene expression changes at different times after Urd supplementation of pyrimidine-starved seedlings were mapped to major pathways of nucleotide metabolism, in order to better understand potential coordination of pathway activities, at the level of transcription. Repression of *de novo* synthesis genes and induction of intracellular and extracellular salvaging genes were early and sustained responses to pyrimidine limitation. Since *de novo* synthesis is energetically more costly than salvaging, this may reflect a reduced energy status of the seedlings, as has been shown in recent studies for seedlings growing under pyrimidine limitation. The unexpected induction of pyrimidine catabolism genes under pyrimidine starvation may result from induction of nucleoside hydrolase *NSH1* and repression of genes in the plastid salvaging pathway, diverting uracil (Ura) to catabolism. Identification of pyrimidine-responsive transcription factors with enriched binding sites in highly coexpressed genes of nucleotide metabolism and modeling of potential transcription regulatory networks provided new insights into possible transcriptional control of key enzymes and transporters that regulate nucleotide homeostasis in plants.

## 1 Introduction

Nucleotides play essential roles in the metabolism of all living organisms. They are the building blocks of nucleic acids, function in energy metabolism and cell signaling cascades, and serve as activated intermediates in carbohydrate metabolism (Bar-Peled and O’Neill, 2011) and other diverse processes (Zrenner et al., 2006; Ashihara et al., 2020; Pietrowska-Borek et al., 2020; Witte and Herde, 2020). Previous biochemical and molecular studies have defined metabolic pathways and genes encoding most enzymes involved in pyrimidine and purine nucleotide metabolism in plants (Wagner and Backer, 1992; Zrenner et al., 2006; Ashihara et al., 2020; Witte and Herde, 2020). These pathways comprise *de novo* synthesis, salvaging of preexisting nucleobases or nucleosides, nucleotide synthesis, and catabolism. In addition, nucleobase, nucleoside and nucleotide transporters move these intermediates within and between cells and tissues (Girke et al., 2014 **;** Major et al., 2017).

Coordination between major pathways of nucleotide metabolism has been further characterized through metabolic profiling studies (Ashihara et al., 2020) and functional studies with mutants. For example, antisense-mediated knockdown of UMP synthase expression in the *de novo* pyrimidine synthesis pathway in potato tubers resulted in increased expression of genes involved in pyrimidine salvaging, increased salvaging pathway activities, and increased levels of uridine nucleotides as a compensatory response (Geigenberger et al., 2005). Similar results were obtained for Arabidopsis RNAi lines with decreased expression of aspartate transcarbamoylase (Chen and Slocum, 2008), which regulates *de novo* pyrimidine pathway activities (Schröder et al., 2005; Bellin et al., 2021; Bellin et al., 2023a). Increased expression of pyrimidine salvaging pathway genes and decreased expression of catabolic pathway genes in response to pyrimidine limitation suggested similar compensatory responses that could help maintain pyrimidine homeostasis in these plants. Thus, nucleotide metabolism responds dynamically to changes in pyrimidine synthesis, at least in part, through transcriptional regulation of enzyme activities. Mechanisms by which this occurs have been poorly investigated.

Here, we explored transcriptional control of nucleotide metabolism in Arabidopsis seedlings using genome-wide expression profiling to investigate changes in expression of genes encoding all known enzymes and transporters of nucleotide metabolism in response to changes in pyrimidine availability. Treatment of seedlings with *N*-(phosphonacetyl)-L-aspartate (PALA), a potent and specific inhibitor of aspartate transcarbamoylase (ATC) and *de novo* pyrimidine synthesis (Bassett et al., 2003; Bellin et al., 2021), resulted in severe growth inhibition and developmental arrest of seedlings within a few days of germination. Normal development was restored by supplementation with exogenous uridine (Urd), which is taken up and used to synthesize pyrimidine nucleotides *via* salvage pathway activities (Witte and Herde, 2020). Expression profiling at various times after Urd addition, and mapping of gene expression changes to metabolic pathways, was a highly informative approach to understanding transcriptional regulation of key enzymes and transporters involved in nucleotide homeostasis. We also examined broader effects of this perturbation in nucleotide metabolism on other cellular processes and identified transcription factors (TF) that may participate in transcription regulatory networks controlling these processes. This represents the first comprehensive study of this type, and it has provided important new insights into the regulation of nucleotide metabolism in plants.

## 2 Materials and methods

### 2.1 Growth of Arabidopsis cultures and experimental treatments

**Figure 1:**
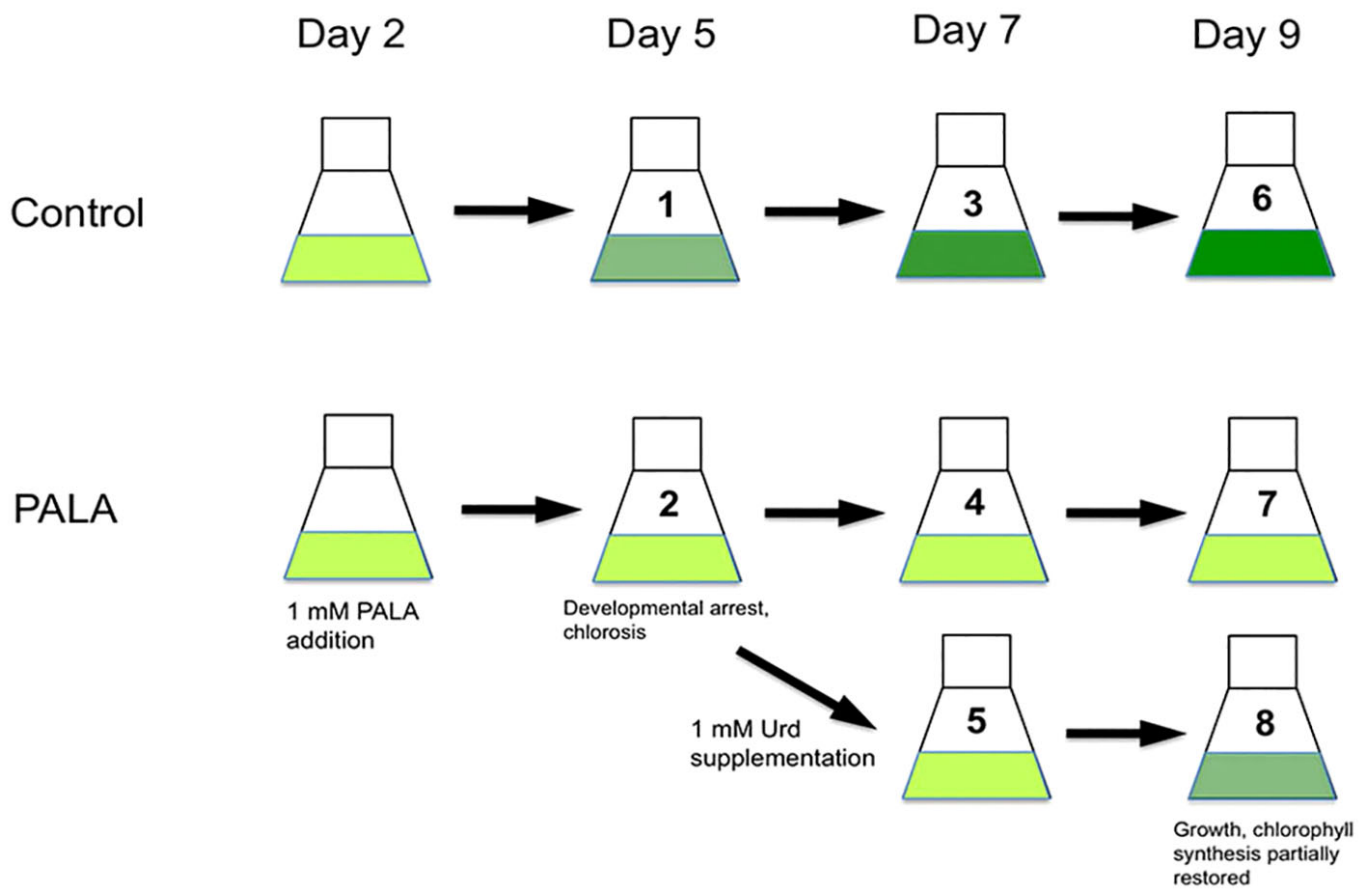
Experiment design. At day 2, PALA was added to a final concentration of 1 mM. Control and PALA-treated seedlings were sampled at days 5, 7, and 9. Some cultures containing 1 mM PALA were supplemented with 1 mM Urd at day 5, then seedlings were sampled at day 7 and day 9. For qRT-PCR assays, samples were also collected at the day 5 and 7 time points, and also the day 12 time point (not shown). Developmental and biochemical phenotype and gene expression data were based upon comparisons of PALA-treated, or PALA+Urd supplemented seedlings with untreated control seedlings at each time point.

*Arabidopsis thaliana* L. seeds (Col-0, Columbia ecotype; stock number CS6673, Arabidopsis Biological Resource Center, Columbus, OH, USA) were surface-sterilized, and approximately 25 seeds were placed into liquid cultures (100 mL of ½× MS medium containing 2.5 mM KH_2_PO_4_ and 1% (w/v) sucrose in a 250-mL flask). The medium contained supplemental phosphate so that this nutrient was not limiting for nucleotide metabolism during the course of the experiment. After 2 days at 4°C to synchronize seed germination, flasks were placed on a rotary shaker at 100 rpm under fluorescent lights (approximately 100 µE m^−2^ s^−1^) and a 16L/8D photoperiod at 25°C. The experiment design is shown in **Figure 1**. PALA (*N*-(phosphonoacetyl)-L-aspartate, NSC #224131-F/30; Developmental Therapeutics Program, National Cancer Institute, NIH, Bethesda, MD, USA) was added to 1 mM final concentration to some of the cultures at day 2. Untreated and 1 mM PALA-containing cultures were harvested 6 h into the light cycle on days 5, 7, 9, and 12. Uracil (Ura), uridine (Urd), and thymidine (Thd) (Sigma-Aldrich; St. Louis, MO, USA) were added to 1 mM final concentrations in some PALA-containing cultures on day 5. Seedlings were harvested on days 7, 9, and 12. Data are representative of two separate experiments, except for gene expression data, which were derived from the second experiment only.

### 2.2 Determination of tissue chlorophyll contents

Seedlings were weighed, homogenized in 2 mL of 95% (v/v) methanol, then centrifuged at 16,000 × *g*, 10 min. The pellet was reextracted with an equal volume of solvent. Total chlorophyll (*a*+*b*) was determined using the method of Arnon (1949) and expressed on a fresh weight basis. Three biological replicates of 8–10 seedlings each were assayed for each treatment.

### 2.3 Catabolic pathway activity assay

Day 9 seedlings were removed from liquid culture, briefly rinsed in pH 5.8 potassium phosphate buffer, then blotted dry and weighed. Two seedlings were placed in a capped 75 × 12-mm plastic tube containing 1 mL of the same buffer containing 1 mM unlabeled Ura + 1 µCi [2-^14^C]-Ura (7.4 GBq mmol^−1^; Sigma-Aldrich, St. Louis, MO, USA). Following a 16-h incubation at 25°C, seedlings were removed from the tube, rinsed in buffer + 1 mM unlabeled Ura, and homogenized in 1 mL of 10% (v/v) perchloric acid (PCA). PCA-soluble and -insoluble fractions were separated by centrifugation at 16,000 × *g*. PCA-insoluble materials were hydrolyzed overnight in 6 M HCl and the supernatant was collected after 16,000 x *g* centrifugation. Soluble and insoluble fractions were neutralized with KOH, and ^14^C-label was quantified by scintillation counting. In parallel, a CO_2_ capture assay was performed to measure Ura catabolism. Seedlings were placed in tubes containing the same reactants as previously described and 6-mm paper discs saturated with 2 M KOH were mounted inside the tube on a plugged 22-gauge syringe needle, inserted through the tube cap. After the incubation period, the assay was terminated by addition of 1 mL of 10% PCA. The tube was re-capped and any additional release of ^14^CO_2_ was captured for 6 h. After drying, ^14^C-label on paper discs was counted directly. Four biological replicates of each assay were used.

### 2.4 Seedling DNA and RNA contents

Seedling DNA contents were determined using a DAPI assay, as described by Baer et al. (1982). Seedling total RNA was isolated using the Plant RNeasy kit (Qiagen, Valencia, CA), according to the manufacturer’s protocol. RNA samples were treated using a TURBO DNA-Free DNase I kit (Thermo Fisher Scientific, Carlsbad, California) to remove genomic DNA contamination. RNA was quantified by A_260_ using a NanoDrop One spectrophotometer.

### 2.5 qRT-PCR analyses of gene expression

Primer3 was used to design primers to Arabidopsis coding sequences. Unique primer pairs generating 100–150-bp amplicons with T_m_ = 60.0 ± 2.0°C were designed for each of the target genes and tested for self-complementarity characteristics. For target genes producing alternatively spliced transcripts, primers were designed to amplify all transcripts associated with the gene. Primers were also designed for the five spiking controls and four invariant control genes (see below). Primer sequences and product sizes for all genes are listed in **Supplemental Table 1**. Primers were synthesized by Operon (Eurofins Genomics, Louisville, KY).

Total RNA (1 µg) was reverse-transcribed (SuperScript III First-Strand Synthesis System; Invitrogen, Carlsbad, CA, USA). Spiking controls (*Bacillus subtilis* transcripts) were added to each sample before cDNA synthesis (per µg total RNA: *lysA*, 0.00002 fmol; *pheB*, 0.0002 fmol; *thrC*, 0.002 fmol; *trpF*, 0.02 fmol; *dapB*, 0.2 fmol). PCR primers were arrayed with sample sets in 384-well optical plates in 10-µL reactions containing 2.5 µL of 0.8 mM primers, 2.5 µL of 1:12.5-diluted sample cDNA, and 5 µL of 2× SYBR Green PCR Master Mix. The master mix and plates were purchased from ABI (Applied Biosystems Inc., Foster City, CA, USA). qPCR analyses were conducted using an ABI Prism 7800HT Sequence Detection System and the SDS v. 2.2.1 software package. PCR amplification steps: 50°C, 2 min; 95°C, 10 min [45 cycles: 95°C, 15 s; 60°C, 1 min]. SYBR Green fluorescence was measured at a threshold value of 0.2, and results were expressed as the C_T_ value. Dissociation curve analyses confirmed amplification of homogeneous targets. qPCR products were sequenced to confirm target specificity. PCR amplification efficiencies for each template (three biological replicates, two technical replicates of each) were determined using the LinRegPCR v 7.5 analysis package (Ramakers et al., 2003). qBase v. 1.3.5 analysis software (Hellemans et al., 2008) was used to calculate normalized relative quantities (NRQ) from raw expression data (C_T_ values) with gene-specific efficiency correction and sample-to-sample interrun calibration (IRC) using four reference genes (AT4G26410, AT4G27960, AT5G15710, AT5G46630; Czechowski et al., 2005). Stability of the reference genes under experimental conditions was evaluated using the geNorm algorithm (Vandesompele et al., 2002) within qBase. Expression data for experimental samples were calculated as ratios ± S.E, relative to control samples. Expression data for day 12 seedlings was gathered only for genes analyzed by qRT-PCR.

### 2.6 RNA-seq analyses

Total RNA sample quality was analyzed using a Agilent Bioanalyzer 2100 platform and prepared for sequencing using a TruSeq cDNA library prep kit (Illumina). Using an Illumina HiSeq 2000 sequencer, an average 53.9 million reads (50 bp, unpaired) per each biological replicate (n = 2) were obtained. Sequencing was performed by Cornell Weill Medical College genomics core (https://research.weill.cornell.edu/core-facilities/genomics). CLC Genomics Workbench v. 10.0.1 software was used to trim and map reads to exons in the Arabidopsis reference genome (TAIR10.1) using Araport11 annotation (Cheng et al., 2017) and 94.8% of reads mapped to exons and introns. Data set normalization, quality control analyses, and gene differential expression (DE) analyses using the R Bioconductor module DESeq2 were performed according to Veerappa et al. (2019). Differentially expressed genes were defined as those having fold-change values of ≥1.5 (padj ≤ 0.05) in response to treatments, relative to untreated controls. RNA-seq reads data have been deposited in the NCBI Sequence Read Archive (BioProject accession #PRJNA704993). Normalized gene expression data were visualized, hierarchically clustered (1-Pearson, average linkage) and further analyzed using Morpheus (https://software.broadinstitute.org/morpheus/). qRT-PCR expression analyses for 35 genes were used to verify RNA-seq expression data for day 5 and day 7 samples (**Supplemental Figure 1**).

### 2.7 Transcription factor enrichment analyses and modeling of transcriptional regulatory networks

ATTED-II (Obayashi et al., 2018) was used to identify the 300 most highly coexpressed Arabidopsis genes with input lists of DE genes functioning in *de novo* synthesis, NTP synthesis, salvaging, catabolism, or nucleobase transport. The PlantRegMap transcription factor (TF) enrichment tool (Tian et al., 2020) was used to identify TF with statistically overrepresented targets in coexpressed genes that were also DE in response to PALA treatment at any time point (day 5, 7, or 9). Lists of significantly enriched TF (*q* ≤ 0.05) were further limited to those TF that were also DE in response to PALA treatment.

iRegNet (Shim et al., 2021) was used to suggest transcriptional regulatory networks beyond potential direct regulations identified in target genes. This resource was queried with TF that were induced or repressed by PALA treatment in day 5 seedlings and which also had enriched targets within the highly co-expressed nucleotide metabolism gene cohorts described above (Bonferroni-corrected *p*-values ≤ 1e−10; 1,000-bp upstream and 1,000-bp downstream flanking sequences of genes were analyzed). iRegNet was also used to explore potential upstream regulons for individual TF and small groups of co-regulated genes.

### 2.8 Construction of metabolic pathways for Arabidopsis nucleotide metabolism

Metabolic pathways and enzyme and transporter annotation and genes for Arabidopsis nucleotide metabolism were adopted from Zrenner et al. (2006) and Witte and Herde (2020). Pathways described in the present study are also supported by information retrieved from AraCyc (https://biocyc.org/group?id=:ALL-PATHWAYS&org-id=ARA#%20).

## 3 Results

### 3.1 The pyrimidine starvation phenotype in Arabidopsis seedlings is reversed by salvaging of exogenous uridine

**Figure 2:**
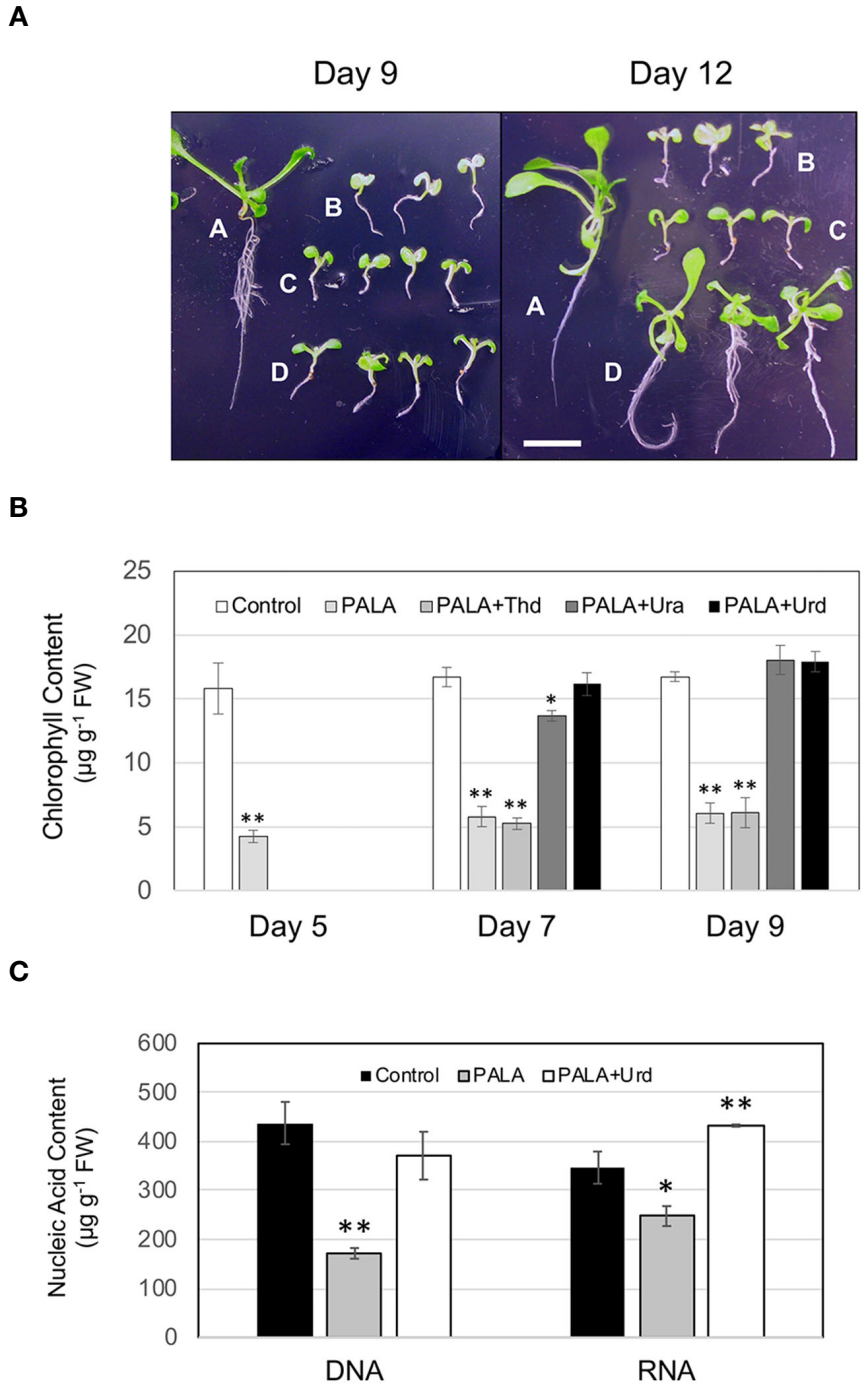
Developmental and biochemical phenotypes resulting from changes in pyrimidine availability in Arabidopsis seedlings. **(A)** Effects of PALA ± supplements on seedling growth and development; control **(A)**, 1 mM PALA **(B)**, 1 mM PALA supplemented with either 1 mM Ura **(C)** or 1 mM Urd **(D)** on day 5, then grown for an additional 4 days (day 9) or 7 days (day 12). Scale bar = 0.5 cm. **(B)** Chlorophyll contents of seedlings growing for 5, 7, or 9 days in control medium, or medium containing 1 mM PALA or 1 mM PALA supplemented with 1 mM Thd, 1 mM Ura, or 1 mM Urd on day 5. **(C)** DNA and RNA contents of Arabidopsis seedlings grown in control medium or 1 mM PALA for 9 days, or in 1 mM PALA for 5 days, then supplemented with 1 mM Urd for four additional days. Values are means ± S.E. for three biological replicates. Asterisks mark samples that are significantly different from the untreated control at each timepoint (*p ≤ 0.05; ** *p* ≤ 0.01).

The experiment design for investigating developmental and biochemical phenotypes related to changes in pyrimidine availability is shown in **Figure 1**. Treatment with 1 mM PALA beginning at 2 days after germination resulted in developmental arrest of seedlings by day 5 and continued through day 9 and day 12 time points (**Figure 2A**). Root growth was especially stunted. Seedlings exhibited severe chlorosis, with chlorophyll contents only 25%–30% of that in untreated control seedlings at days 5, 7, and 9 (**Figure 2B**). At day 9, seedling DNA and RNA contents were 60% and 30% lower than controls, respectively (**Figure 2C**). This pyrimidine starvation phenotype persisted at day 12, indicating that metabolism of PALA was negligible.

Addition of 1 mM Urd to day 5 cultures containing 1 mM PALA reversed seedling developmental arrest beginning around 9, when increased hypocotyl and primary root growth, expansion of cotyledons, and development of leaves were noted (**Figure 2A**). Chlorophyll contents were not significantly different from control seedlings by day 7 (**Figure 2B**). By day 9, DNA contents were the same as controls and RNA contents were 20% higher than in control seedlings (**Figure 2C**). In cultures grown for an additional 3 days (day 12, 7 days of Urd supplementation), seedling development was nearly indistinguishable from control seedlings at the day 9 stage, including significant lateral root development and two sets of rosette leaves (**Figure 2A**). In contrast, development of seedlings supplemented with 1 mM Ura was not different from PALA-treated seedlings in day 9 or day 12 cultures. However, at day 7, chlorophyll contents of these seedlings were 80% of those in control seedlings and were not different from controls at day 9 (**Figure 2B**), suggesting a limited ability to salvage this nucleobase. Seedlings in thymidine-supplemented cultures (not shown) did not look different than PALA-treated seedlings at either day 9 or day 12 (**Figure 2A**). Chlorophyll contents were also not different from those of PALA-treated seedlings, indicating little salvaging of this nucleoside (**Figure 2B**).

### 3.2 Increased uracil catabolism explains poor salvaging of this nucleobase in pyrimidine starved seedlings

**Figure 3:**
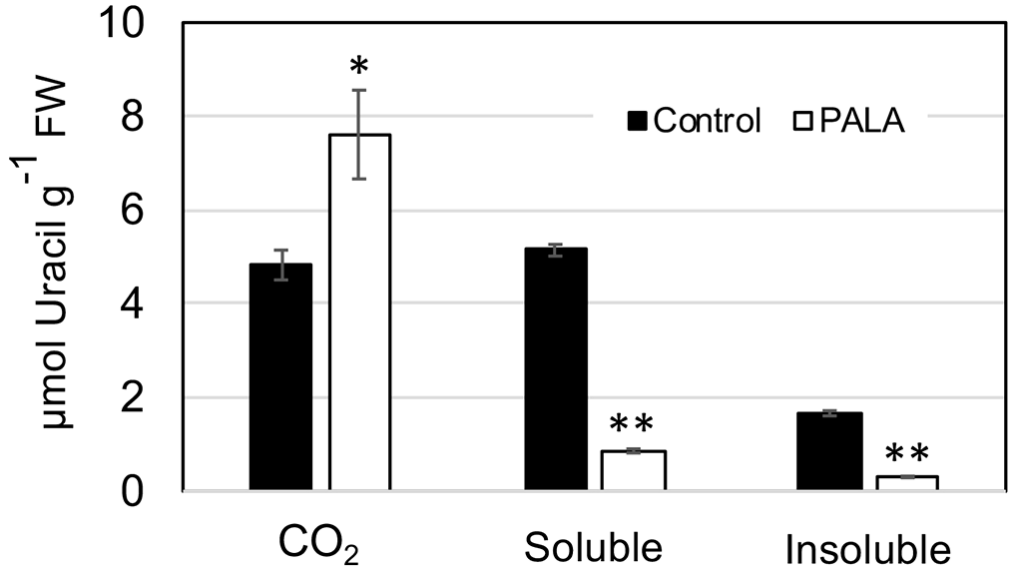
Metabolism of [2-^14^C]-uracil in 9-day-old PALA-treated seedlings. The soluble fraction is free uracil or labeled metabolites in seedling tissues. The insoluble fraction represents incorporation of uracil label into nucleic acids. CO_2_ is label released by uracil catabolism. Asterisks mark samples that are significantly different from the control (*p ≤ 0.05; ** *p* ≤ 0.01).

The limited salvaging of Ura in PALA-treated day 9 seedlings was supported by assays of [2-^14^C]-uracil metabolism (**Figure 3**). In untreated control seedlings, approximately 40% of the label taken up by the plant was recovered as ^14^CO_2_, a measure of catabolic pathway activity. This was similar to the catabolic activity previously reported for WT Arabidopsis seedlings (Zrenner et al., 2009). PALA treatment increased catabolism of Ura twofold, with decreased labeling of soluble metabolite and insoluble nucleic acid fractions (**Figure 3**). Interestingly, PALA-treated seedlings took up only 75% as much labeled Ura as control seedlings, which may reflect a reduced energy status in their tissues.

### 3.3 Genome-wide transcriptional profiling reveals biological processes impacted by changes in pyrimidine availability

RNA-seq identified 11,845 unique genes that were differentially expressed (DE) at one or more stages of seedling development (day 5, day 7, day 9) in response to PALA treatment, with or without Urd supplementation. **Table 1** summarizes the number of induced and repressed DE genes in response to different experimental treatments at different time points. Remarkably, most genes that remained DE in response to PALA at day 9 were no longer DE after 4 days of Urd supplementation, relative to untreated control seedlings. This condition paralleled the recovery of seedling development.

**Table 1:** Numbers of induced and repressed DEG in Arabidopsis seedlings in response to PALA treatment, with or without Urd supplementation at day 5, day 7, and day 9 time points.

Comparison groups	Relative expression (fold change)		Number of DEGs		
**Day 5 control vs. PALA**	UP	≥2×	1,369	2,365	4,528
		1.5–2×	996		
	DOWN	1.5–2×	1,254	2,163	
		≥2×	909		
**Day 7 control vs. PALA**	UP	≥2×	2,781	4,081	8,816
		1.5–2×	1,300		
	DOWN	1.5–2×	2,506	4,735	
		≥2×	2,229		
**Day 7 control vs. PALA+Urd**	UP	≥2×	930	1,663	3,284
		1.5–2×	733		
	DOWN	1.5–2×	1,301	1,621	
		≥2×	320		
**Day 9 control vs. PALA**	UP	≥2×	1,735	2,541	5,092
		1.5–2×	806		
	DOWN	1.5–2×	904	2,551	
		≥2×	1,647		
**Day 9 control vs. PALA+Urd**	UP	≥2×	25	149	207
		1.5–2×	124		
	DOWN	1.5–2×	47	58	
		≥2×	11		

**Figure 4:**
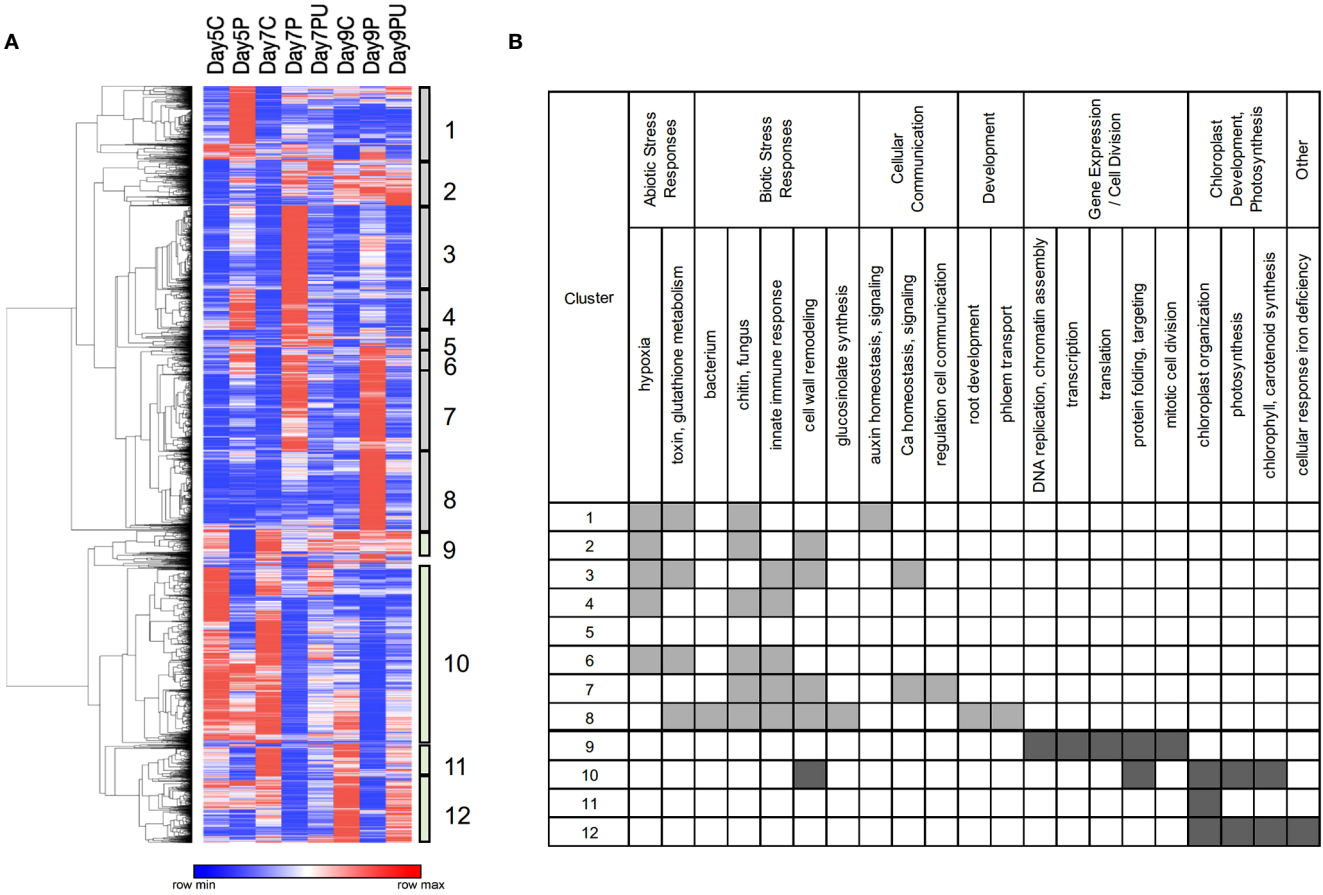
Heatmap showing clustering **(A)** of normalized expression values for 6,245 DE genes (FC ≥ 2). Clusters 1–8 show genes with increased expression in response to PALA treatment, with reversal by Urd supplementation, and represent a timeline of early to late responses (left to right). Genes in clusters 9–12 show genes that were repressed by PALA treatment, with Urd reversal, with early to late responses (left to right). Control, PALA treatment, and PALA + Urd treatment (C, P, PU) at days 5, 7, and 9. Statistically overrepresented GO BioProcess categories for genes in each cluster **(B)**.

Clustering of genes by their overall expression profiles (**Figure 4**) facilitated identification of 12 gene clusters and their enriched GO BioProcess categories that were co-regulated in response to changes in pyrimidine availability over time. Most genes induced by PALA function primarily in abiotic and biotic stress responses. Genes involved in cellular responses to hypoxia or pathogens and in auxin, and Ca^2+^ homeostasis and signaling were regulated beginning at day 5. Genes regulating root development were not induced until day 9, although inhibition of root growth occurred much earlier. In contrast, genes in the cluster 10 expression module were broadly repressed on day 5. Among the genes that belong to cluster 10, many are involved in regulating cell wall loosening during growth, including several expansins and an endoglucanase, and many others that encode proteins which regulate gene expression and cell division activities required for normal seedling growth and development. At day 7, expression of large numbers of genes involved in plastid development and photosynthesis was uniformly decreased. Thus, overall patterns of gene expression supported developmental and biochemical phenotypes but further revealed major abiotic and biotic stress responses resulting from pyrimidine starvation.

### 3.4 Pyrimidine starvation induces genes regulating synthesis of the phytoalexin camalexin

One early response to pyrimidine starvation was the strongly increased expression of genes encoding enzymes that synthesize the indole glucosinolate camalexin, the major phytoalexin in Arabidopsis which plays an important role in defense against pathogens and herbivores (**Supplemental Figure 2**; Lemarié et al., 2015). Several genes in this pathway are regulated by MYB51/HIGH INDOLIC GLUCOSINOLATE 1 (Frerigmann et al., 2015), which was shown to be strongly induced at day 5 (**Supplemental Figure 2**). In contrast, expression of myrosinase 1 and 2, which activate glucosinolates, producing toxic isothiocynante and thiocyanate defense compounds (Shroff et al., 2008), was repressed (**Supplemental Figure 2**). This suggests that this defense response would be ineffective in PALA-treated seedlings, whereas metabolic diversion of L-tryptophan from auxin synthesis (Zhao, 2012) to camalexin synthesis would be expected to impact root development (Overvoorde et al., 2010) and other auxin-regulated processes.

### 3.5 Pyrimidine limitation caused reduced expression of genes involved in chloroplast development and photosynthetic pigment synthesis

The severe chlorosis associated with pyrimidine limitation in the light-grown seedlings was previously noted. Genes in chlorophyll (**Supplemental Figure 3**) and carotenoid (**Supplemental Figure 4**) synthesis pathways were coordinately downregulated by PALA treatment by day 7, although genes involved in plastid organization were repressed earlier, beginning on day 5. Protochlorophyllide reductase A (PORA), which functions in phototransformation of protochlorophyllide to chlorophyllide (Liu et al., 2017) was repressed nearly 40-fold in the tetrapyrrole synthesis pathway. Genes encoding MEP pathway enzymes and prenyltransferase activities synthesizing the isoprenoid geranylgeranyl diphosphate (GGPP) precursor for phytyl diphosphate, the phytol tail donor to chlorophyllide *a* in the final step of chlorophyll *a* synthesis (Kim et al., 2013; Kopcsayová and Vranová, 2019), were also repressed. PALA induction of chlorophyll catabolism enzymes chlorophyllase-2 and the magnesium dechelatase STAY-GREEN1 (Hörtensteiner and Kräutler, 2011) on day 5 preceded repression of chlorophyll synthesis genes beginning on day 7 (**Supplemental Figure 3**) and likely contributed to decreased chlorophyll contents of these seedlings. Expression of GATA1, GATA2, and GLK2, transcription factors which activate genes promoting chlorophyll biosynthesis and photosynthetic membrane organization (Liu et al., 2017; Zubo et al., 2018) was repressed (**Supplemental Figure 3**). Additionally, numerous genes encoding chlorophyll *a/b* binding proteins of photosystems I and II were repressed (data not shown). For example, *LHCB2.4* transcript levels continued to decrease in response to PALA treatment to only 5% of control levels by day 12, but Urd supplementation on day 5 restored levels of this transcript to 60% of those in control seedlings by day 12 (**Supplemental Figure 1**).

Carotenoids are also essential photosynthetic pigments which are synthesized from GGPP and repression of isoprenoid pathway genes would limit their production, in addition to the synthesis of isoprenoid-derived hormones abscisic acid and gibberellins (Kopcsayová and Vranová, 2019), which regulate a wide range of physiological and developmental processes. Phytoene synthase, which regulates carotenoid synthesis (Stanley and Yuan, 2019), and other pathway genes were further repressed in response to pyrimidine limitation (**Supplemental Figure 4**).

### 3.6 Genes encoding enzymes and transporters of nucleotide metabolism are responsive to changes in pyrimidine availability

**Supplemental Table 2** lists 152 genes encoding enzymes or transporters involved in nucleotide metabolism and the co-regulated Arg synthesis pathway. qRT-PCR expression analyses (**Supplemental Figure 1**) verified the expression of 35 individual genes at day 5 and day 7 time points but also included day 12 expression data, which are not represented in transcriptome datasets. Day 12 expression data identified genes that responded more slowly to changes in pyrimidine availability. In most cases, RNA-seq and qRT-PCR expression data were highly similar.

Among the 2,163 genes that were downregulated by PALA on day 5 but not at later stages, genes in the GO BioProcess category “*de novo*” pyrimidine nucleobase biosynthetic process (GO: 0006207) were the most enriched, although *de novo* purine synthesis genes were also repressed (**Supplemental Table 2**). Approximately half of the genes involved in nucleotide metabolism and Arg synthesis were DE in response to PALA treatment and expression profiles for most of these genes were reversed by Urd supplementation. Genes that function in salvaging of extracellular nucleotides were strongly induced by pyrimidine starvation, but, unexpectedly, pyrimidine and purine catabolic pathway genes were also induced under limited pyrimidine availability.

**Figure 5:**
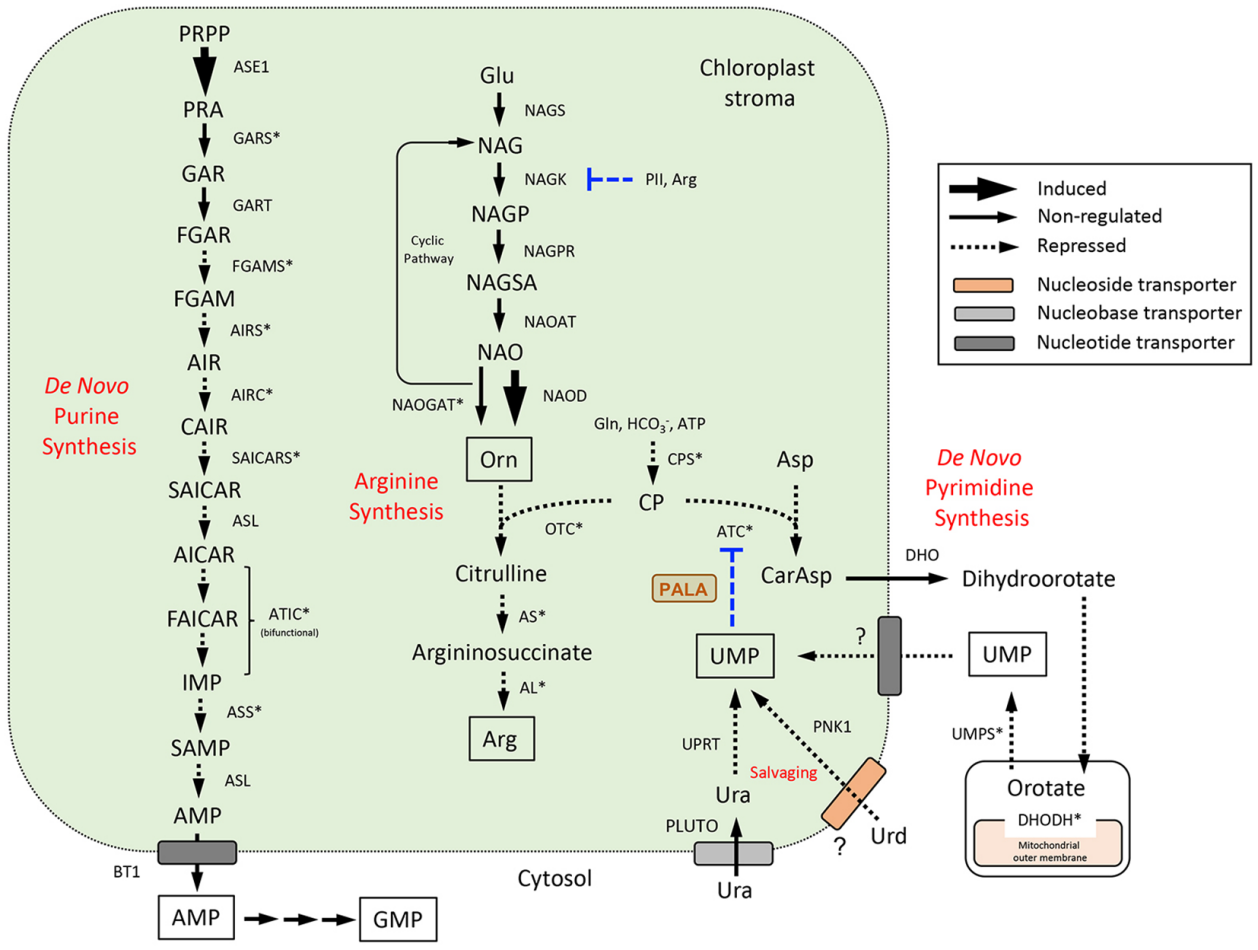
*De novo* purine and pyrimidine synthesis pathways and their subcellular compartmentation in Arabidopsis (Zrenner et al., 2006; Witte and Herde, 2020). Pyrimidine synthesis is integrated with the synthesis of Arg at the level of CP utilization by ATC and OTC. The UMP end product exerts major control over pyrimidine synthesis through allosteric regulation of ATC activity (Belin et al., 2020) and is also formed through uracil salvaging by UPRT or Urd salvaging by PNK1 (see **Figure 6**). UMP and Orn modulate CPS activities; Arg and the N-sensing regulatory protein PII control Arg synthesis at the NAGK step (Slocum, 2005). Gene expression information is integrated into pathways to show steps that are induced or repressed in response to PALA treatment. Highly coexpressed genes in the three pathways are indicated with an asterisk. PALA inhibition of ATC is shown. Abbreviations used for enzymes and transporters are listed in **Supplemental Table 2** and pathway intermediates are listed in **Supplemental Table 3**.

**Figure 6:**
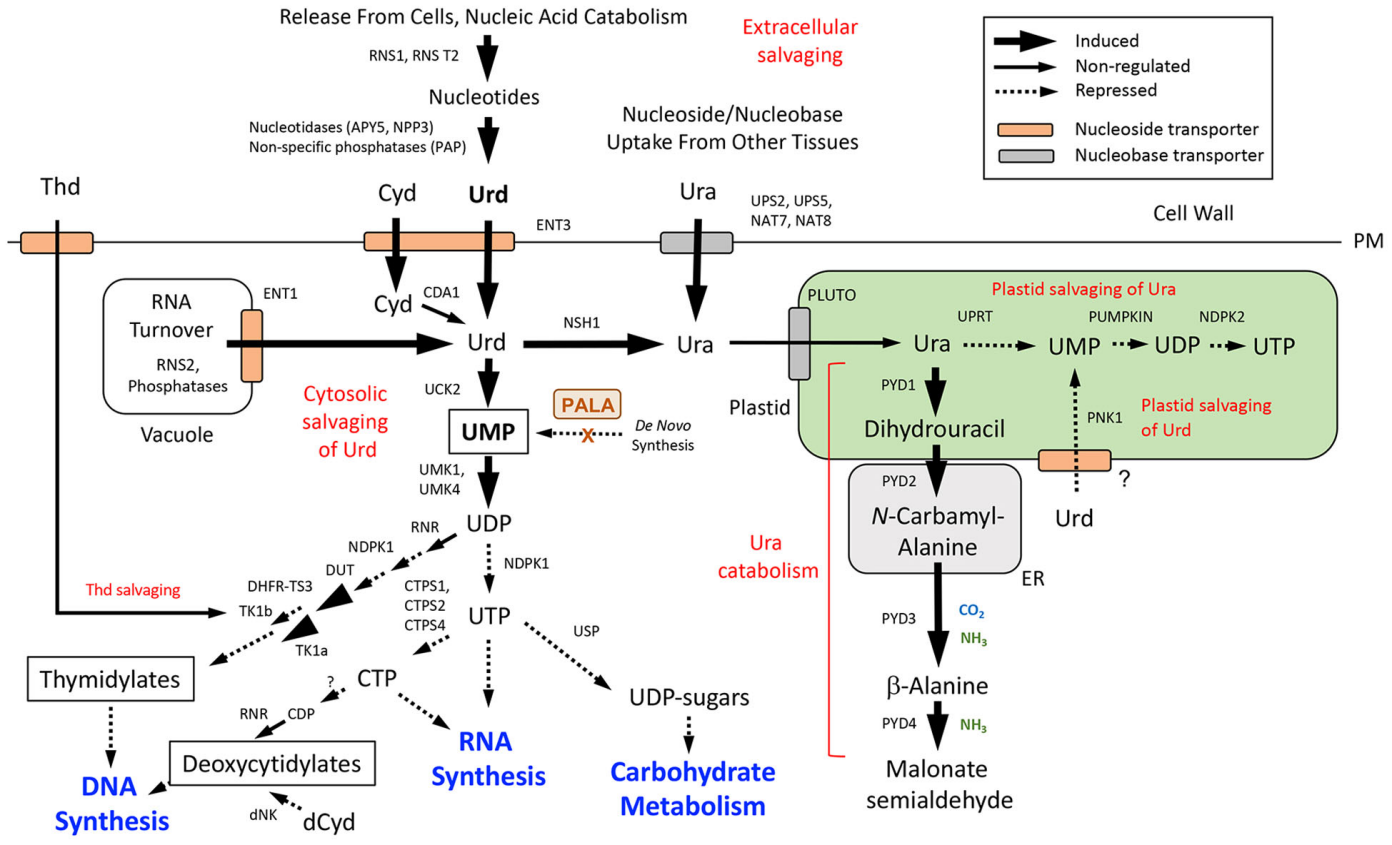
Pyrimidine salvaging (intracellular and extracellular) and catabolism. Pathway organization and subcellular compartmentation are according to Witte and Herde (2020). Gene expression information is integrated into pathways to show steps that are induced or repressed in response to PALA treatment. Salvaging of nucleobases derived from RNA turnover in the vacuole and extracellular sources of nucleotides are indicated. Cytosolic salvaging of Cyd (*via* CDA1 and Urd), Urd, and Thd and plastid salvaging pathways for Ura and Urd are shown. Induced salvaging of Cyd to CMP by UCK2 and downstream cytidylates by UMK1/4 and NDPK1 activities is not shown). Further metabolism of UMP into thymidylate, uridylate, and (deoxy)cytidylate precursors of RNA and DNA synthesis and carbohydrate metabolism are also shown. Inhibition of UMP synthesis by PALA inhibition of the *de novo* pathway is indicated (see **Figure 5**). Abbreviations used for enzymes and transporters are listed in **Supplemental Table 2** and pathway intermediates are listed in **Supplemental Table 3**.

**Figure 7:**
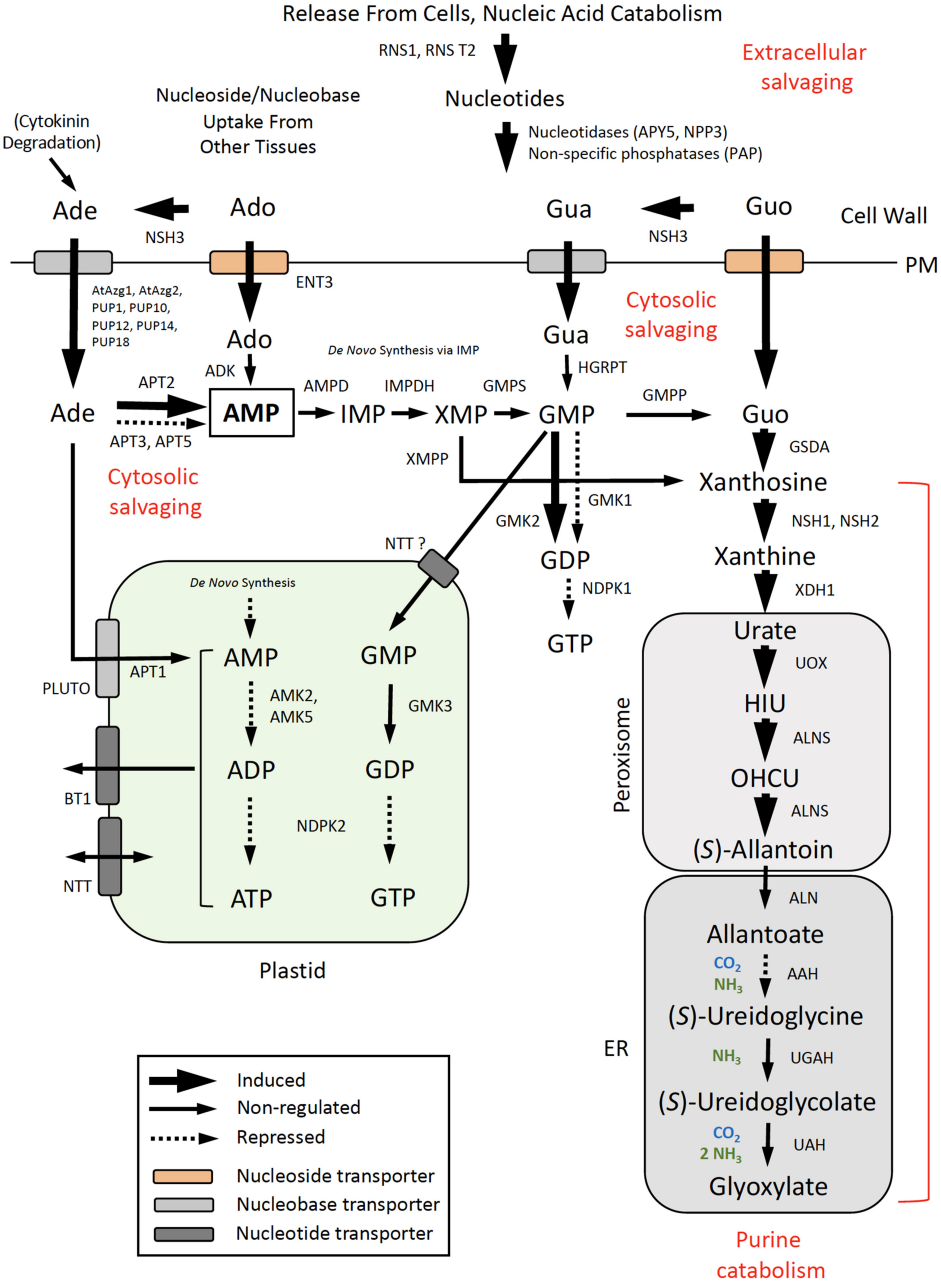
Purine salvaging (intracellular and extracellular) and catabolism. Pathway organization and subcellular compartmentation are according to Witte and Herde (2020). Gene expression information is integrated into pathways to show steps that are induced or repressed in response to PALA treatment. Salvaging of Ado and Gua derived from RNA turnover in the vacuole, deoxypurine metabolism, and salvaging of inosine and hypoxanthine are not shown. Abbreviations used for enzymes and transporters are listed in **Supplemental Table 2** and pathway intermediates are listed in **Supplemental Table 3**.

Expression data for DE genes were mapped to the *de novo* pyrimidine and purine synthesis pathways (**Figure 5**) and pathways for pyrimidine (**Figure 6**) and purine (**Figure 7**) salvaging (intracellular and extracellular) and catabolism to better understand possible metabolic responses to changes in pyrimidine availability, at the level of transcriptional regulation. Abbreviations for enzymes and transporters are provided in **Supplemental Table 2** and abbreviations for pathway intermediates are listed in **Supplemental Table 3**.

#### 3.6.1 *De novo* synthesis pathways and arginine synthesis

Aspartate transcarbamoylase (ATC), the target of PALA inhibition, catalyzes the committed step in the *de novo* synthesis of pyrimidines in the plastid (Bellin et al., 2021); **Figure 5**). The remaining four reactions leading to UMP synthesis occur in the cytosol and mitochondrion inner membrane space and UMP is the precursor for synthesis of other pyrimidine nucleotides (Witz et al., 2012; Witte and Herde, 2020). PALA treatment resulted in decreased expression of CPSA, ATC, and DHODH in day 5 seedlings but not at later time points (**Supplemental Table 2**). PALA decreased UMPS expression in day 7 seedlings, but this response was not observed when day 5 seedlings were supplemented with Urd for 2 days.

**Figure 5** also shows the pathway for arginine (Arg) synthesis, which is functionally coupled to *de novo* pyrimidine synthesis at the level of CP utilization by both OTC and ATC (Slocum, 2005). OTC initiates Arg synthesis from Orn, which is usually synthesized from Glu using a cyclic pathway in which the acetyl moiety is conserved (Slocum, 2005; Winter et al., 2015). PALA did not change the expression of any genes encoding the five enzymes in Orn synthesis, although it did increase expression of the NAOD gene in day 9 seedlings (**Table S2**). This enzyme catalyzes Orn synthesis with metabolism of the acetyl group. Interestingly, expression of the PII sensory protein which activates NAGK was decreased in day 7 seedlings, a response which was partially reversible by Urd. PII exerts major metabolic control of Orn and Arg synthesis *via* Arg end-product inhibition (Ferrario-Méry et al., 2006). Further Arg synthesis from Orn would likely be downregulated since expression of the three enzymes catalyzing these steps (OTC, AS, AL) was repressed. Depression of Arg synthesis would divert CP to *de novo* pyrimidine synthesis, perhaps decreasing PALA inhibition of ATC by competing for substrate binding sites in the enzyme.

The 12-step plastid-localized pathway for *de novo* synthesis of the purine AMP is shown in **Figure 5**. AMP is exported to the cytosol for further metabolism to other purine nucleotides (Zrenner et al., 2006; Witte and Herde, 2020; see **Figure 7**, below). Expression of most of these genes was decreased by PALA treatment, except for ASE1, and was reversible by Urd (**Supplemental Table 2**). Co-expression analyses for genes in the *de novo* pyrimidine and purine synthesis pathways and Arg synthesis pathway (ATTEDII) confirmed that many are coordinately regulated (**Figure 5**).

#### 3.6.2 Pathways for pyrimidine salvaging and catabolism

Pathways for the synthesis of pyrimidine nucleotides by salvaging of pyrimidine nucleosides and nucleobases, and competing pathways for their catabolism, are shown in **Figure 6**. Under pyrimidine limitation, UMP may be synthesized directly from Urd by UCK2, which was induced by PALA at day 5 and later stages (**Supplemental Table 2**). An important source of Urd is the vacuole, where ribonuclease RNS2 facilitates RNA turnover and acid phosphatases further metabolize ribonucleotide products. Urd and Cyd are then exported to the cytosol *via* the tonoplast transporter ENT1, where these nucleosides can be salvaged to UMP and other pyrimidines (Bernard et al., 2011; Hillwig et al., 2011; Witte and Herde, 2020). Both RNS2 and ENT1 were induced by PALA at day 5 but not at later time points, suggesting that changes in activities of this salvaging pathway were an early response to pyrimidine starvation.

Extracellular pyrimidine nucleosides may also be produced by degradation of RNA by RNases and further metabolism of nucleotides by phosphatases (Chen et al., 2000; Robinson et al., 2012), as occurs in the vacuole. Cyd from intracellular or extracellular sources would be metabolized by CDA1, producing Urd (Chen et al., 2016). Although CDA1 transcripts were not detected in RNA-seq analyses (**Supplemental Table 2**), strong CDA1 induction by PALA and repression by Urd was observed in qRT-PCR assays (**Supplemental Figure 1**). PALA treatment upregulated expression of plasma membrane (PM)-localized ENT3 (Girke et al., 2014) on day 5, and cell-wall-localized ribonuclease RNS1 was among the most strongly upregulated genes at all time points. Extracellular phosphatase NPP3 and apyrases APY5 and APY6, which may function as ecto-phosphatases that regulate extracellular NTP (eNTP) levels (Pietrowska-Borek et al., 2020; Clark et al., 2021) also exhibited strong, early induction by PALA (**Supplemental Table 5**). Since no PM-localized transporter is known to take up nucleotides directly into plant cells (Major et al., 2017), nucleotide metabolism to nucleosides, or further metabolism to nucleobases, is required for intracellular salvaging to occur (Riewe et al., 2008). Expression of extracellular salvaging genes did not increase when PALA-treated seedlings were supplemented with Urd.

Early induction of genes involved in both intracellular and extracellular salvaging of pyrimidine nucleosides suggest that these processes are highly responsive to changes in pyrimidine availability in the cell. However, expression profiles for genes involved in further metabolism of UMP into uridylates, (deoxy)cytidylates and thymidylates, and downstream processes like nucleic acid synthesis and carbohydrate metabolism (**Figure 6**) suggest that these processes were repressed, consistent with biochemical data, such as decreased nucleic acid contents of PALA-treated seedlings (**Figure 2C**). For example, increased expression of UMK1 and UMK4 in response to PALA does not occur until day 7 (**Supplemental Table 2**), potentially limiting the flux of salvaged UMP into pyrimidine nucleotide pools during early stages of pyrimidine starvation. Downregulation of NDPK1 and CTPS1 would limit UTP availability for RNA synthesis and carbohydrate metabolism. DNA synthesis would be inhibited by decreased DUT expression and increased expression of DHFR-TS3, which inhibits activities of DHFR-TS1 and DHFR-TS2 in thymidylate synthesis (Gorelova et al., 2017). Increased cytosolic thymidine kinase TK1a expression at day 5 could facilitate salvaging of Thd to dTMP, although further incorporation into thymidylates would be inhibited by decreased NDPK1 expression (**Supplemental Table 2**).

In addition to cytosolic salvaging of Urd, plastid salvaging of Urd is also possible. Recently, Chen et al. (2023) characterized PLASTID NUCLEOSIDE KINASE 1 (PNK1) as an inosine kinase which also functions as a Urd kinase *in vivo*. In PALA-treated seedlings, PNK1 expression decreased at day 7 and was reversible by Urd supplementation (**Supplemental Table 4**). Uptake of Urd into the plastid implies the existence of an unknown nucleoside transporter in this organelle.

Salvaging of Ura is also possible. Ura can be taken up directly by several PM-localized nucleobase transporters, such as NAT8, whose expression was strongly upregulated by PALA beginning on day 7 (**Supplemental Table 2**). Cytosolic metabolism of Urd to Ura can also occur as a result of nucleoside hydrolase NSH1 activity (Jung et al., 2009) and *NSH1* was induced late, on day 9. After uptake into the plastid by the nucleobase transporter PLUTO, Ura is successively metabolized to UMP, UDP, and UTP *via* UPRT, a plastid UMP kinase (PUMPKIN) and NDPK2 (Witz et al., 2012). The plastid salvaging pathway is distinct from the *de novo* synthesis of UMP, which is initiated in the plastid but completed in the cytosol and mitochondrion (**Figure 5**; Witz et al., 2012). It is also distinct from the UMP product of Urd salvaging in the plastid (Chen et al., 2023). Expression of plastid salvage pathway genes was strongly downregulated by PALA at days 7 and 9 and was reversed by Urd. PLUTO expression was unusual in that it did not change in response to PALA but was downregulated at day 7 by Urd supplementation.

Alternatively, Ura in the plastid may be catabolized by PYD1 to dihydrouracil, which is further metabolized outside of the plastid by PYD2, PYD3, and PYD4, recycling pyrimidine N as NH_3_ into general N metabolism (Zrenner et al., 2009). Catabolic pathway genes were upregulated by PALA and this response was reversed by Urd supplementation (**Supplemental Table 2**). Increased catabolism of labeled Ura in PALA-treated seedlings (**Figure 3**) provided biochemical evidence in support of gene expression data.

#### 3.6.3 Pathways for purine salvaging and catabolism

Pathways for purine salvaging and catabolism are shown in **Figure 7**. As for pyrimidine metabolism, coordinated induction of vacuolar RNS2(+phosphatases)/ENT1 and extracellular RNS1(+phosphatases)/ENT3 genes at day 5 suggests that RNA and nucleotide degradation would provide Ado and Guo for salvaging or catabolism as an early response to pyrimidine limitation. Increased expression of cell wall-localized, purine-specific nucleoside hydrolase *NSH3* (Jung et al., 2011) would facilitate metabolism of the extracellular purine nucleosides to the corresponding nucleobases, Ade and Gua, for uptake by several PM-localized, nucleobase transporters. PALA increased *NSH3* expression more than twofold on day 7, but Urd did not reverse this response. Expression of PUP1 and other purine permeases was strongly upregulated by PALA beginning on day 5. In contrast, expression of PUP4 and AtAzg2 transporters increased at day 7 in response to Urd supplementation but not PALA treatment alone. Expression of adenine phosphoribosyltransferase APT2, which metabolizes Ade to AMP, was similarly regulated by Urd but not by PALA, at day 7, but its expression increased at day 9 in PALA-treated seedlings. In contrast, adenosine kinase (ADK1, ADK2; **Supplemental Table 5**) was not regulated by PALA or Urd. Thus, salvaging of Ade, rather than Ado, may represent the main salvaging activity for adenylates, in response to altered pyrimidine metabolism.

Genes encoding enzymes for the *de novo* synthesis of GMP from AMP in the cytosol, *via* the IMP intermediate (**Figure 7**), or HGRPT, which salvages Gua directly, were not regulated in response to PALA treatment. Downregulation of *AMK2*, *AMK5*, and *NDPK2* suggests that further metabolism of AMP (derived from *de novo* synthesis or Ade/Ado salvaging) or GMP into adenylate or guanylate pools in the plastid would be inhibited. Although cytosolic GMK2 was induced on day 7, NDPK1 expression decreased on day 5, suggesting that GTP synthesis from GDP would also be limited.

The pathway for purine catabolism is also shown in **Figure 7**. Xanthosine cannot be salvaged (Yin et al., 2014) so its formation resulting from GSDA deamination of Guo (Dahncke and Witte, 2013) or dephosphorylation by an XMP phosphatase (XMPP; Heinemann et al., 2021) commits it to the catabolic pathway. Most AMP is degraded *via* GMP, which is metabolized to Guo by an unknown GMP phosphatase (Witte and Herde, 2020). Although *GSDA, XDH1*, and *ALNS* expression was induced on day 5, *NSH1* and *NSH2* expression increased later, at day 9, which might limit purine catabolism, since the NSH1/NSH2 complex has been shown to constitute the major nucleoside hydrolase activity that degrades xanthosine (Baccolini and Witte, 2019). Decreased expression of *AAH* at day 9 may further limit purine degradation and recycling of purine N to general N metabolism.

### 3.7 Transcription factors responsive to pyrimidine starvation function mainly in regulation of abiotic and biotic stress responses

**Figure 8:**
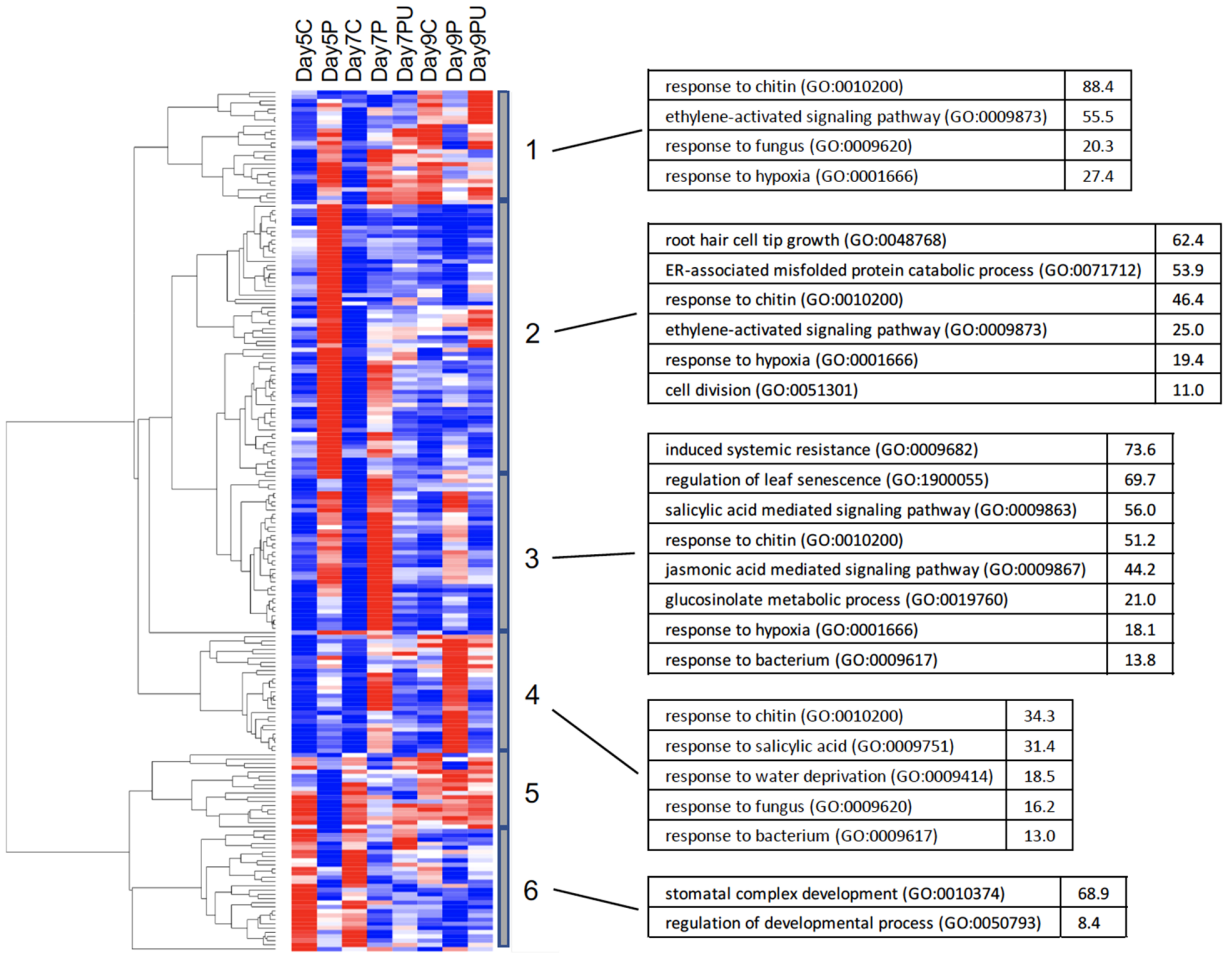
Heatmap showing clustering of normalized expression values for 89 DE TF genes (FC ≥ 2). Clusters 1–4 show genes with increased expression in response to PALA treatment, compared with untreated controls. Urd supplementation reversed this response. Genes in clusters 5–6 show the opposite response to PALA and Urd. Control, PALA treatment and PALA + Urd treatment (C, P, PU) at days 5, 7, and 9. Highly overrepresented GO BioProcess categories and fold-enrichment values for each cluster are shown.

Gene expression profiling for transcription factors (TF) which were DE in response to PALA treatment is shown in **Figure 8**. As in **Figure 4**, clustering revealed early (day 5) and later (day 7, 9) responses and associated enriched GO BioProcess categories. Most induced TF genes in clusters 1–4 are involved in regulating abiotic and biotic stress responses, consistent with the functional characterization of early regulated genes (**Figure 4**) and included numerous WRKY and ethylene-responsive TF. Early, repressed TF mainly regulate developmental processes.

### 3.8 Identification of potential transcriptional regulatory networks for nucleotide metabolism in Arabidopsis

We identified TF with enriched targets (Tian et al., 2020) within individual cohorts of highly coexpressed genes representing the major metabolic pathways of nucleotide metabolism and Arg synthesis. Enriched TF and their target genes are arrayed in **Supplemental Table 4**, and annotation and expression data for these TF are given in **Supplemental Table 5**. Many of the co-regulated genes function in different pathways and have common TF targets that may serve to coordinate nucleotide metabolism at the level of transcription. Induction of a large number of ERF TF is correlated with repression of genes involved in nucleotide synthesis and Arg synthesis, and induction of nucleobase transporter genes. Targets for induced WRKY TF are strongly overrepresented in CPSA, whose repression would decrease synthesis of carbamoyl-P, further limiting pyrimidine and Arg synthesis.

**Figure 9:**
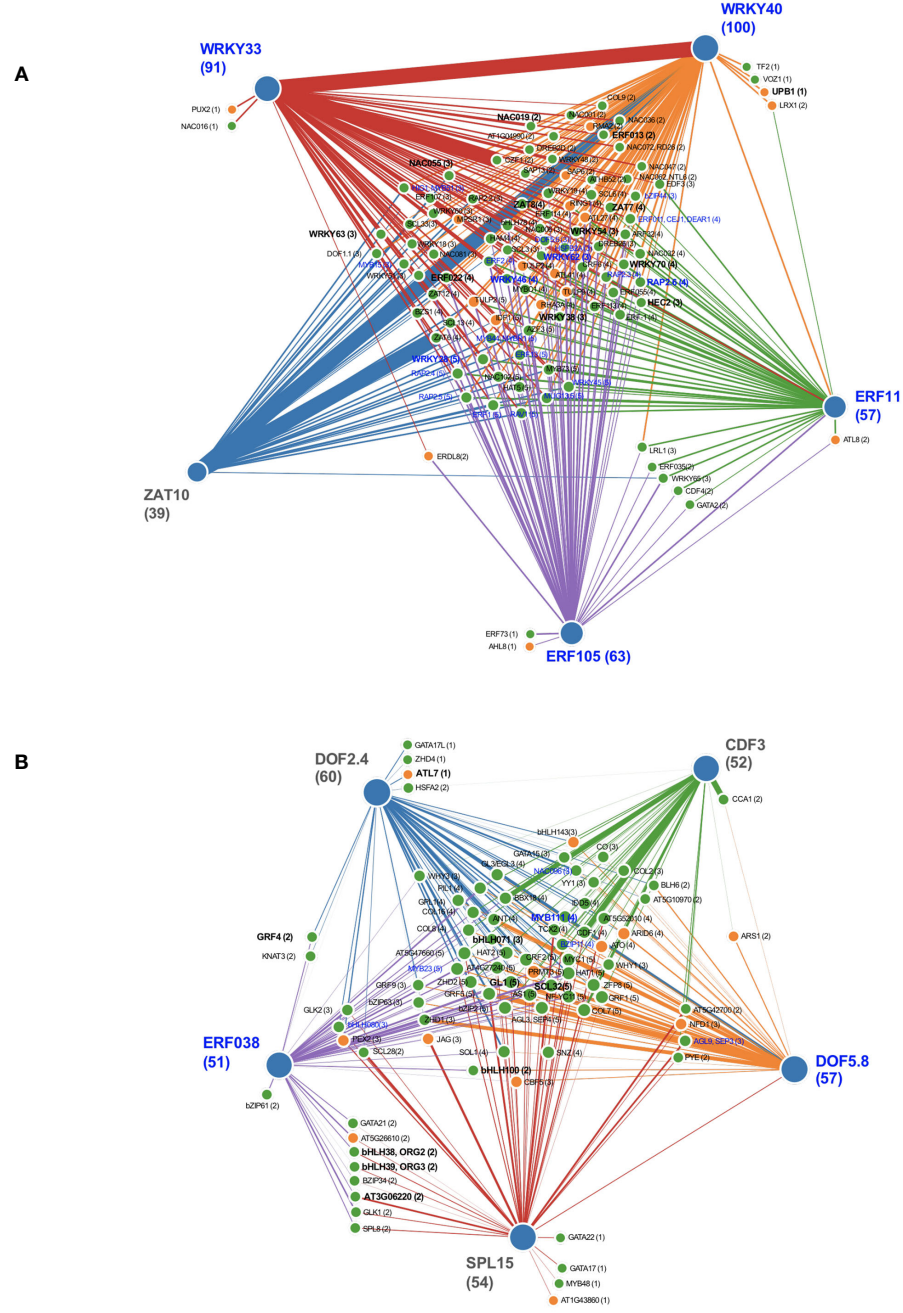
Integrative network graphs (iRegNet) showing five highly coexpressed upstream genetic regulators (outer nodes) for differentially expressed TF (FC ≥ 1.5) that were either induced **(A)** or repressed **(B)** by pyrimidine starvation at day 5. Edge thickness indicates strength of co-expression between genes. Induced genes with FC ≥5 on day 5 and repressed genes with FC ≥ 5 on day 7 (bold font) represent the most highly regulated early TF genes. TF with enriched targets in genes of nucleotide metabolism, representing potential downstream regulations, are indicated (blue font).

To better understand transcriptional control of nucleotide metabolism, we modeled transcriptional regulatory networks for TF whose induction or repression by PALA on day 5 was reversed by Urd. These TF represented the earliest responses to changes in pyrimidine availability. iRegNet (Shim et al., 2021) was used to generate regulatory network graphs for day 5 responses (**Figure 9**). This resource identifies significantly overrepresented upstream regulators of query genes, integrating gene-to-gene co-expression data to generate realistic regulatory networks. As is shown in **Figure 9A**, nearly all TF induced by PALA on day 5 are potentially regulated by one or more upstream transcription factors ERF11, ERF105, ZAT10, WRKY33, and WRKY40. Transcription factors repressed by PALA treatment are downstream of ERF039, SPL15, DOF2.4, DOF5.8, and CDF3 (**Figure 9B**).

**Figure 10:**
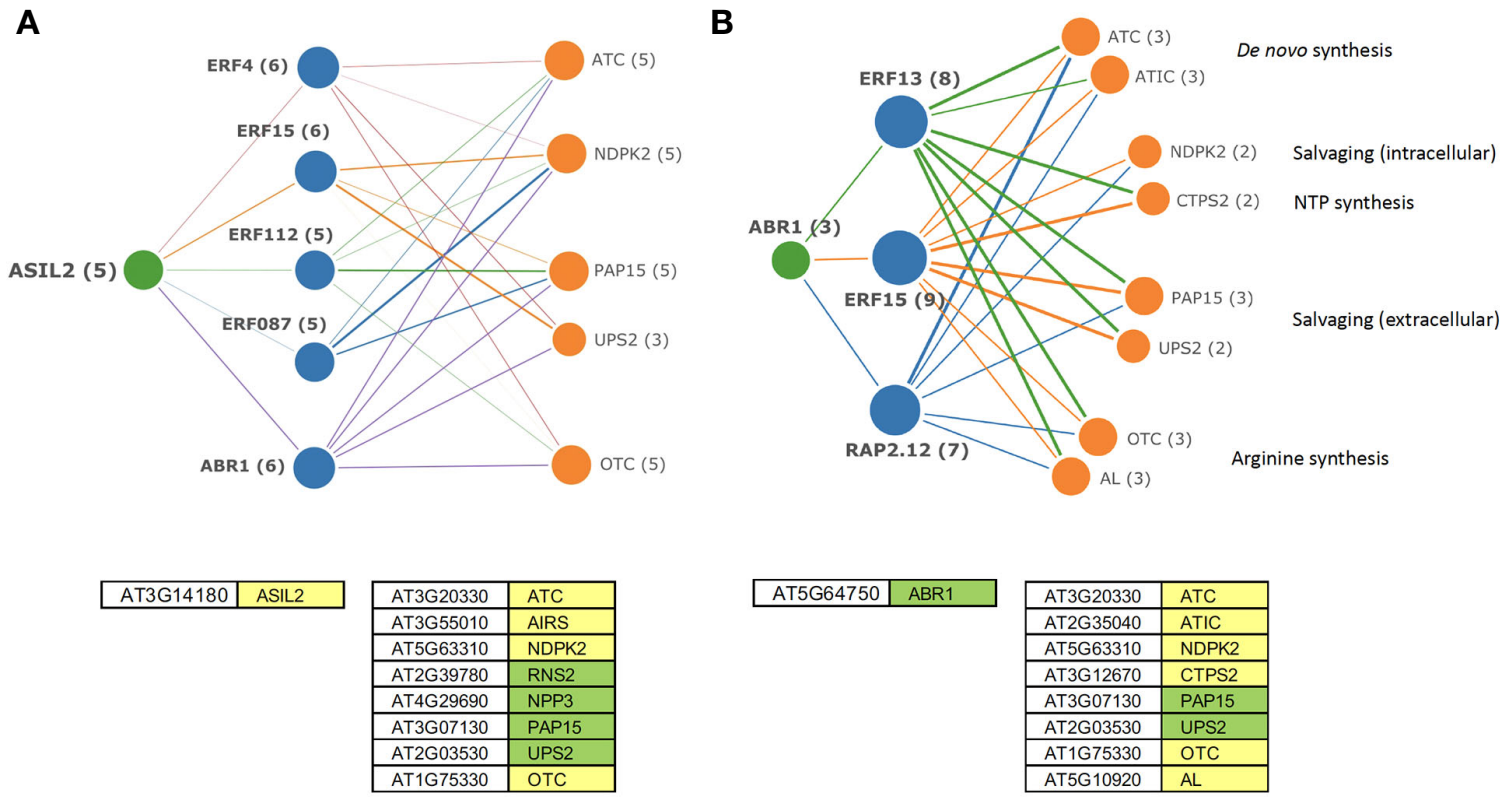
Hypothetical regulatory networks for transcription factors ASIL2 **(A)** and ABR1 **(B)**. Edge thickness indicates strength of co-expression between genes. ASIL2 is repressed on day 5. ABR1, a potential downstream target of ASIL2 is induced by day 7. Both TF have binding sites within the nucleotide metabolism genes and may further regulate the downstream targets by induction of ethylene-responsive TF, coordinating synthesis, and salvaging of nucleotides with Arg synthesis. Models were developed based on the DE genes with statistically enriched targets for ASIL2 and ABR1 (AGI numbers; green—induced genes, yellow—repressed genes).

Some of the predicted upstream regulators of DE TF are themselves regulated by PALA ± Urd treatments. Several have binding sites that are enriched within co-regulated genes of nucleotide metabolism and, thus, have potential for direct regulations of those genes. Many of the potentially regulated downstream TF in the network graphs also have enriched targets in nucleotide metabolism genes. This information can be used to construct hypothetical regulons for individual genes or groups of genes that may help coordinate metabolic responses to altered pyrimidine levels in seedlings. For example, ASIL2 has binding sites in genes that regulate *de novo* nucleotide synthesis, intracellular and extracellular salvaging, and Arg synthesis activities (**Supplemental Table 2**) and is repressed by PALA at day 5. A hypothetical transcriptional regulatory network for ASIL2 is shown in **Figure 10A**. In this model, induction of ethylene-responsive TF, including ABR1, with targets in the same nucleotide metabolism genes, could further coordinate their expression. Additional direct and indirect regulations involving ABR1 are shown in **Figure 10B**. These network graphs also validate TF enrichment analyses (**Supplemental Table 4**) as a general approach to identifying nucleotide metabolism genes that are likely to be co-regulated by individual TF.

**Figure 11:**
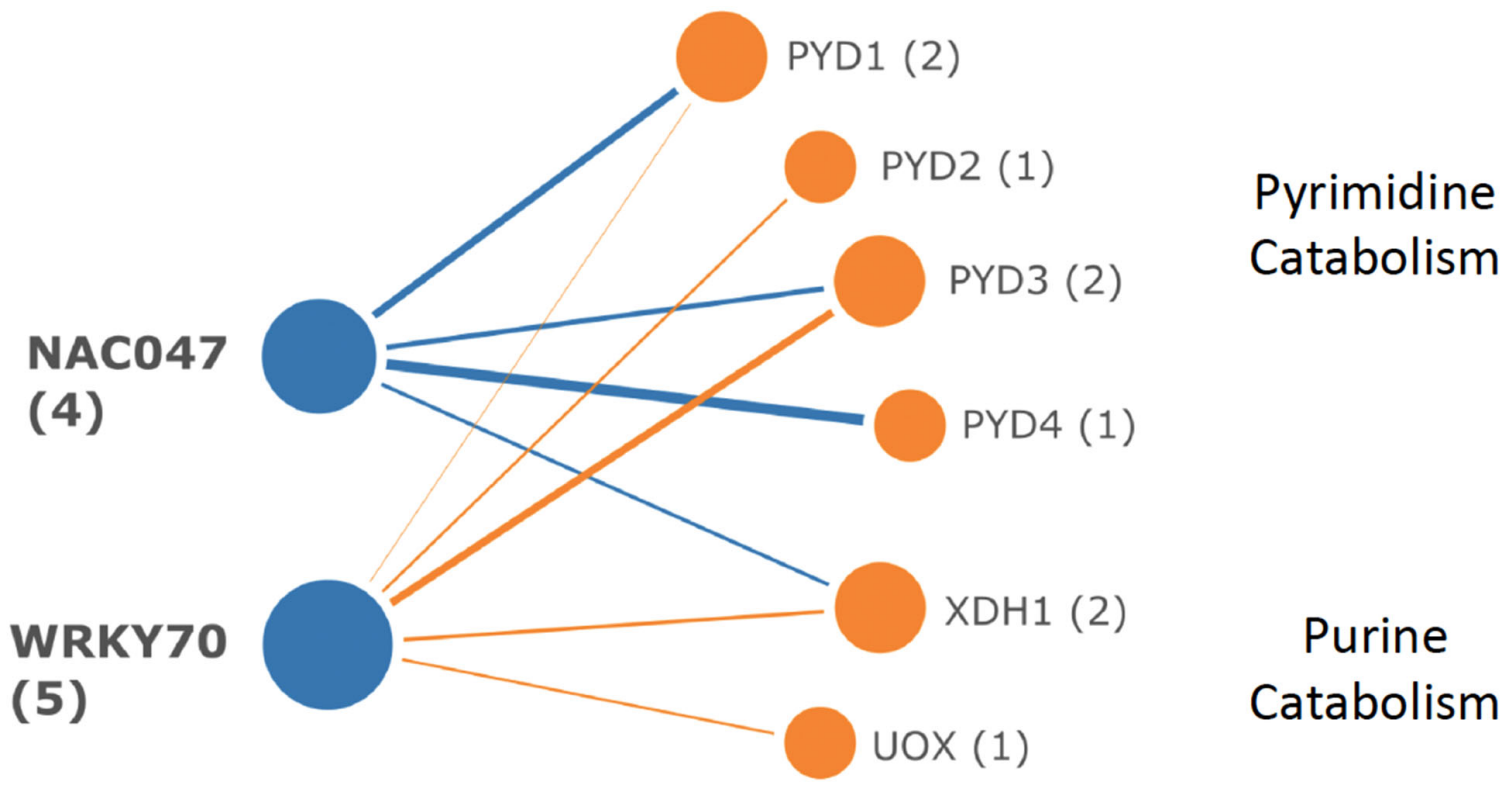
Hypothetical regulon for DE genes of pyrimidine and purine catabolism. Transcription factors WRKY70 and NAC047 are highly induced by PALA and could coordinately regulate target gene expression in both pathways, although binding sites for these TF are not statistically overrepresented in the target genes.

Attempts to model similar regulatory networks for DE catabolic pathway genes and TF with enriched targets in those genes were unsuccessful. Using all DE catabolic genes (**Supplemental Table S2**) as queries in iRegNet, the hypothetical regulon shown in **Figure 11** was produced. In this model, highly induced WRKY70 and NAC047, which function in abiotic and biotic stress responses, would activate genes of pyrimidine and purine catabolism.

## 4 Discussion

PALA inhibition of pyrimidine synthesis and Urd supplementation offered a useful model for investigating the effects of altered pyrimidine availability on biochemical, developmental, and transcriptome-level responses in Arabidopsis seedlings.

Pyrimidine starvation led to developmental arrest in seedlings 5 days after germination, when seed stores of pyrimidines were exhausted. Gene expression profiling suggests a nearly complete collapse of anabolic activities and increased catabolic activities in these seedlings, paralleled by general abiotic and biotic stress responses. One interesting stress response was the strong induction of indole glucosinolate synthesis genes, which may divert L-tryptophan from the synthesis of auxin (Zhao, 2012), an important regulator of root development (Overvoorde et al., 2010). Repression of genes involved in auxin signaling or regulation of auxin homeostasis was one of the earliest responses to PALA treatment. Early inhibition of root growth in pyrimidine starved seedlings was observed in the present study and was also reported in a previous investigation (Schröder et al., 2005).

A general repression of genes involved in chloroplast development and photosynthetic pigment synthesis paralleled strongly decreased chlorophyll contents in PALA-treated seedlings. Impaired plastid functions have been reported in a variety of plant mutants with defects in nucleotide metabolism (Sugimoto et al., 2007; Lange et al., 2008; Mainguet et al., 2009; Chen et al., 2015; Ye et al., 2016; Chen et al., 2018; Ohler et al., 2019) and would significantly impact energy metabolism and major metabolic pathways, including nucleotide synthesis, salvaging, and catabolism activities that reside in this organelle. Bellin et al. (2023b) recently reported a low photosynthetic efficiency and increased production of reactive oxygen species in the ATC knockdown line *atc#1*, which exhibits reduced pyrimidine levels. Additionally, other genes of central energy metabolism (glycolysis, TCA cycle, respiration) were significantly repressed, relative to the Col-0 WT.

Genome-wide gene expression profiling in Arabidopsis seedlings supports some general observations regarding possible transcriptional control of nucleotide metabolism in this plant. In response to pyrimidine starvation, salvaging pathways (intracellular and extracellular) were upregulated and *de novo* synthesis was downregulated. Synthesis of nucleotides by salvaging of preformed nucleobases and nucleosides requires less energy than *de novo* synthesis (Zrenner et al., 2006). Thus, salvaging would be more efficient under conditions of impaired energy metabolism resulting from defects in chloroplast development and photosynthesis, or other energy-generating processes. Ohler et al. (2019) reported that UCK1/UCK2 are responsible for most pyrimidine salvaging *in vivo* and *uck1/uck2* mutants exhibit severe growth inhibition. This suggests that *de novo* pyrimidine synthesis activities alone are insufficient to support normal seedling growth and development (Chen and Thelen, 2011). While knockouts of any genes in the *de novo* pyrimidine pathway are lethal (Schröder et al., 2005), it is clear that salvaging activities alone were able to sustain normal seedling growth and development in the present study, under continued strong inhibition of *de novo* synthesis by PALA. It is interesting to note that moderate genetic repression of each gene in the *de novo* pathway did not produce visible phenotypes in the solanaceous plants tobacco and potato (Schröder et al., 2005). Stronger inhibition of pathway activities was required to significantly reduce nucleotide pool sizes, and this resulted in growth inhibition without large changes in the developmental program, although roots were more sensitive than shoots to pyrimidine limitation. They concluded that the plants compensated for pyrimidine starvation with reduced growth and that even reduced nucleotide pools could sustain basic metabolic processes (Schröder et al., 2005). Strong inhibition of ATC with 1 mM PALA resulted in severe growth inhibition during the first few days after germination, with developmental arrest by day 5, suggesting that nucleotide pools were insufficient to maintain seedling growth. Bassett et al. (2003) had previously shown that growth inhibition of Arabidopsis seedlings on medium containing 0.1 mM PALA occurred much more slowly, reaching developmental arrest by day 12 after germination. The higher PALA concentration used in the present study allowed for transcriptome analyses during early stages of pyrimidine salvaging and recovery of seedling growth and development in a timeframe in which nutrient limitation would not become a factor in the analyses.

It is remarkable that Urd supplementation reversed transcriptome-level responses within the first 2 days. After 4 days, Urd salvaging restored expression of most genes to control levels, in spite of continued PALA inhibition of *de novo* synthesis in these plants, and normal seedling development resumed. Very little is known about mechanisms for sensing nucleotide pool sizes in plant tissues, but it seems clear that they respond quickly and dynamically, even to major perturbations in nucleotide metabolism. Beyond direct Urd uptake from surrounding plant tissues, it seems clear that extracellular salvaging activities that metabolize nucleic acids and nucleotides, and parallel activities in the vacuole, involving RNA turnover, are tightly coupled to intracellular salvaging activities and would play important roles in salvaging during normal plant growth.

An important finding of this study is that *de novo* pyrimidine and arginine synthesis pathway activities are likely coordinated at the level of transcriptional regulation, in addition to the well-characterized metabolic control of CP allocation to each pathway (Slocum, 2005). Feedback control of ATC and *de novo* pyrimidine synthesis by UMP is well known (Bellin et al., 2021), but the mechanism by which this might occur *in vivo* has been poorly understood, since UMP synthesis in this pathway occurs outside of the plastid (Witz et al., 2012). In contrast, UMP synthesis in the plastid, resulting from Urd salvaging by PNK1 (Chen et al., 2023) or Ura salvage pathway activities (Witz et al., 2012), may regulate ATC activity, thus providing a mechanism to balance salvaging and *de novo* synthesis activities. The observed co-expression of *de novo* pyrimidine and purine synthesis pathway genes was also seen in the present study and may be an important mechanism for regulating pool sizes for these nucleotides.

Induction of pyrimidine catabolism genes, under conditions of severe pyrimidine limitation, was unexpected in PALA-treated seedlings. In two previous studies with ATC knockdown lines with less severe phenotypes (Chen and Slocum, 2008; Bellin et al., 2023a), catabolic pathway genes were repressed. In the present study, enhanced catabolism of labeled Ura in day 9 seedlings may have resulted from repression of Ura salvaging pathway genes, leading to Ura accumulation in the plastid and increased availability to PYD1 and the catabolic pathway. Failure of salvaged UMP/UDP to be further incorporated into downstream nucleotide synthesis (repression of NDPK1, CTPS1 would limit UTP, CTP synthesis) and nucleotide-dependent metabolism, might also favor Urd accumulation in the cytosol where NSH1 would metabolize Urd to Ura for further catabolism in the plastid. The relatively late induction of NSH1 at day 9, and repression of PNK1 at day 7, in PALA-treated seedlings raises an interesting question for further study. Would Urd salvaging have been be less effective if it began on day 9, rather than at the earlier (day 5) time point? Repression of cytosolic NDPK1 and plastid NDPK2 would also limit ATP and GTP synthesis and salvaged AMP/GMP would be channeled toward xanthosine and the catabolic pathway. This might represent a mechanism for balancing purine and pyrimidine catabolism in the seedlings.

Coordination of *de novo* synthesis, salvaging, and catabolic pathway activities is required to balance pyrimidine and purine pool sizes resulting from changing metabolic demands during plant growth and development. However, a recent review of plant nucleotide metabolism (Witte and Herde, 2020) noted the paucity of information regarding transcriptional regulation of these processes. We performed TF enrichment analyses for highly coexpressed genes encoding enzymes and transporters of nucleotide metabolism to identify TF that might be involved in direct regulations of those genes, limiting the analyses to both TF and targets that were DE in response to changes in pyrimidine availability. Further modeling of transcriptional regulatory networks permitted construction of hypothetical ASIL2 and ABR1 regulons that may coordinate key steps in pyrimidine and purine synthesis and salvaging, as well as Arg synthesis. Similarly, WRKY 70 and NAC047 may coordinate expression of pyrimidine and purine catabolism genes. A small number of TF that were strongly induced or repressed by PALA on day 5 may further coordinate downstream TF networks representing nearly all regulated TF with enriched targets in nucleotide metabolism genes. We recognize the limitations of integrating transcriptomic data in systems studies of metabolism (Cavill et al., 2016; Richelle et al., 2019). However, approaches used in the current study may prove useful for further characterizing transcription regulatory networks for plant nucleotide metabolism.

## Data availability statement

The datasets presented in this study can be found in online repositories. The names of the repository/repositories and accession number(s) can be found below: https://www.ncbi.nlm.nih.gov/genbank/, PRJNA704993.

## Author contributions

RS: Conceptualization, Data curation, Formal Analysis, Funding acquisition, Investigation, Methodology, Project administration, Resources, Software, Supervision, Validation, Visualization, Writing – original draft, Writing – review & editing. CP: Formal Analysis, Investigation, Methodology, Writing – review & editing. ZL: Funding acquisition, Methodology, Project administration, Resources, Software, Supervision, Writing – review & editing.
