# Supplementary material for: Transcriptional reprogramming of nucleotide metabolism in response to altered pyrimidine availability in Arabidopsis seedlings
Source: Front Plant Sci. 2023 Nov 2;14:1273235. doi: 10.3389/fpls.2023.1273235 (PMC10652772; doi:10.3389/fpls.2023.1273235)
Supplement: Supplementary file 1 [file DataSheet_1.pdf]

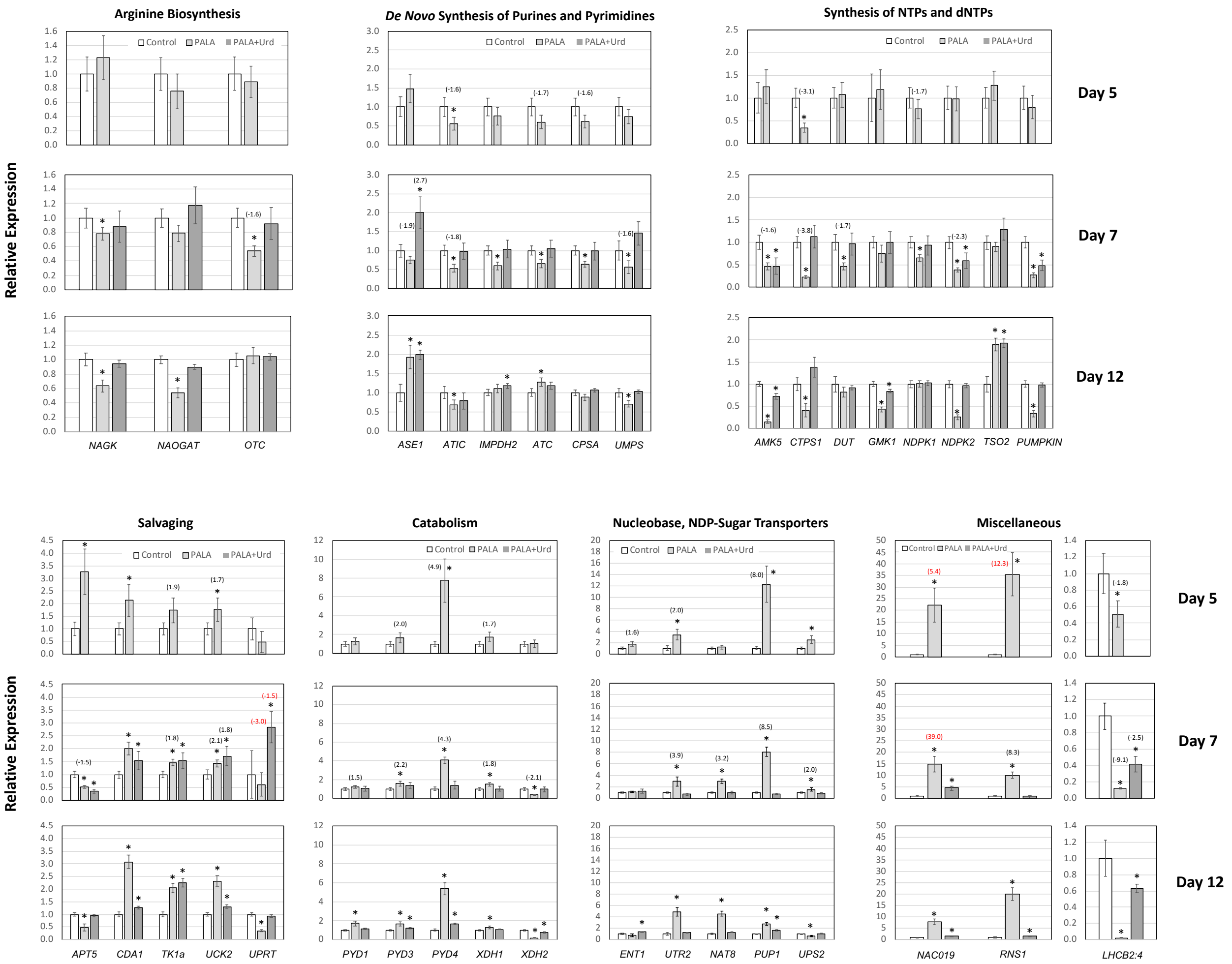

Supplemental Figure 1. qRT-PCR relative expression for nucleotide metabolism genes involved in *de novo* synthesis, NTP synthesis, salvaging, catabolism and transport in Day 5, Day 7 or Day 12 stage seedlings grown in control medium, medium containing 1 mM PALA, or 1 mM PALA supplemented with 1 mM uridine beginning on Day 5. Genes in the arginine synthesis pathway and genes with large PALA-inducible increases (*NAC019*, *RNS1*) or decreases (*LHCB2:4*) in expression are also included. *UTR2* encodes UDP-glucose transporter 2. Gene symbols are listed in Supplemental **Tabl3.** Data represent means ± S.E. for three biological replicates and two technical replicates of each. Asterisks mark samples that are significantly different from the untreated control at each timepoint (**p* ≤ 0.05). Numbers in parentheses are fold-change values based on RNA-seq expression data for differentially-expressed genes, for comparison.

Supplemental Figure 2. Effects of pyrimidine limitation on expression of genes encoding enzymes involved in synthesis and activation of the indole glucosinolate camalexin. Fold-change (FC) values for DE genes are presented as a heatmap at day 5, 7 and 9 time points (C = Control, P = PALA treatment, PU = PALA + Urd treatment). Expression values for untreated controls were the reference at each time point.

AGI	Symbol	Fold-Change (Linear)					Annotation
		Day5 CvP	Day7 CvP	Day7 CvPU	Day9 CvP	Day9 CvPU	
**Regulation of Indole Glucosinolate Synthesis**							
AT1G18570	MYB51/HIG1	4.1	6.6	2.3			Transcription factor MYB51/HIGH INDOLIC GLUCOSINOLATE 1, HIG1
AT1G74080	MYB122		2.7				Transcription factor MYB122
**Camalexin Synthesis from L-Tryptophan**							
AT4G39950	CYP79B2	1.9	3.8	2.0	2.8		Cytochrome P450, CYP79B2; tryptophan N-monooxygenase 1
AT2G22330	CYP79B3		1.9	1.9			Cytochrome P450, CYP79B3; tryptophan N-monooxygenase 2
AT2G30750	CYP71A12		12.9	7.1	12.7		Cytochrome P450, CYP71A12; Indoleacetaldoxime dehydratase
AT2G30770	CYP71A13		6.2	2.1			Cytochrome P450, CYP71A13; Indoleacetaldoxime dehydratase
AT4G30530	GGP1		1.5				Gamma-glutamyl-peptidase 1, GGP1
AT1G74100	SOT16	3.0	3.0	1.8	2.0		Cytosolic sulfotransferase 16; SOT16
AT1G13090	CYP71B15	1.8	1.6				Cytochrome P450 CYP71B15; dihydrocamalexate synthase/camalexin synthase (bifunctional)
AT3G26830	CYP71B15		4.2	2.0			Cytochrome P450 CYP71B15; dihydrocamalexate synthase/camalexin synthase (bifunctional)
**Glucosinolate Activation**							
AT5G26000	TGG1		-2.5	-2.6	-4.2		Myrosinase 1; thioglucoside glucohydrolase 1, TGG1
AT5G25980	TGG2	-3.7	-9.9	-4.9	-11.1		Myrosinase 2; thioglucoside glucohydrolase 2, TGG2

MYB51/HIG1-regulated

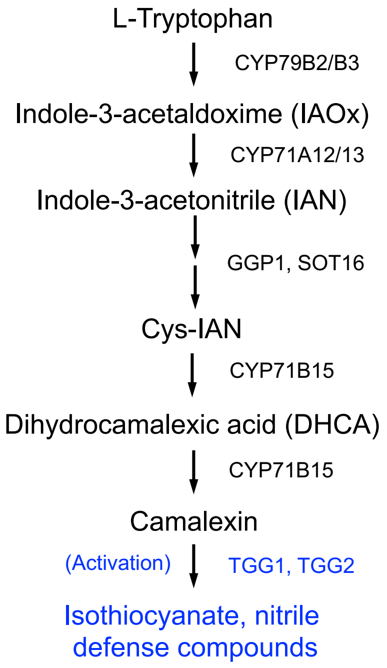

Supplemental Figure 3. Effects of PALA on expression of genes encoding enzymes of chlorophyll synthesis and catabolism and some regulators of chlorophyll synthesis. Fold-change (FC) values for DE genes are presented as a heatmap at day 5, 7 and 9 time points (C = Control, P = PALA treatment, PU = PALA + Urd treatment). Expression values for untreated controls were the reference at each time point. Pathways for chlorophyll biosynthesis (A) and catabolism (B) are shown below. Enzymes catalyzing each step in the pathways figures below are numbered in the table.

AGI Locus	Step	Symbol	Enzyme Name	EC Number	Fold-Change (Linear)				
					Day5 CvP	Day7 CvP	Day7 CvPU	Day9 CvP	Day9 CvPU
**Chlorophyll Synthesis**									
AT5G64050	1	OVA3	Glutamyl-tRNA synthetase	6.1.1.17	-1.8	-2.6	-1.9	-1.5	
AT1G58290	2	HEMA1	Glutamyl-tRNA reductase 1	1.2.1.70				-1.8	
AT1G09940		HEMA2	Glutamyl-tRNA reductase 2						
AT2G31250		HEMA3	Glutamyl-tRNA reductase 3			-2.0			
AT5G63570	3	GSA1	Glutamate-1-semialdehyde aminotransferase 1	5.4.3.8		-1.9	-1.5	-1.5	
AT3G48730		GSA2	Glutamate-1-semialdehyde aminotransferase 2			-3.2	-1.6	-2.5	
AT1G69740	4	HEMB1	Porphobilinogen synthetase 1	4.2.1.24		-1.9		-2.1	
AT1G44318		HEMB2	Porphobilinogen synthetase 2						
AT5G08280	5	HEMC	Porphobilinogen deaminase	2.5.1.61		-3.0	-1.6	-2.6	
AT2G26540	6	UROS	Uroporphyrinogen synthase	4.2.1.75		-2.4	-1.8	-2.1	
AT3G14930	7	HEME1	Uroporphyrinogen decarboxylase 1	4.1.1.37		-3.6	-1.6	-3.9	
AT2G40490		HEME2	Uroporphyrinogen decarboxylase 2			-2.9	-1.8	-3.0	
AT1G03475	8	CPX1	Coproporphyrinogen oxidase 1	1.3.3.3		-2.2	-1.6	-2.1	
AT4G03205		CPX2	Coproporphyrinogen oxidase 2					-2.1	
AT4G01690	9	PPOX1	Protoporphyrinogen oxidase 1	1.3.3.4		-1.9		-2.0	
AT5G14220		PPOX2	Protoporphyrinogen oxidase 2					-1.6	
Heme synthesis branch -->									
AT5G26030		FC1	Ferrochelatase 1	4.99.1.1	1.5	2.0		1.5	
AT2G30390		FC2	Ferrochelatase 2			-2.2	-1.7	-2.4	
	10		Magnesium chelatase	6.6.1.1					
AT1G08520		CHLD	Subunit ChlD			-2.0	-1.5	-2.5	
AT5G13630		CHLH	Subunit ChlH			-1.6		-2.7	
AT4G18480		CHLI-1	Subunit ChlI-1			-2.8	-1.7	-2.8	
AT5G45930		CHLI-2	Subunit ChlI-2			-3.7	-1.9		
AT3G59400		GUN4	Tetrapyrrole-binding protein GUN4		-1.7	-3.0	-1.9	-3.2	
AT4G25080	11	CHLM	Mg-Protoporphyrin IX methyltransferase	2.1.1.11		-2.9	-1.6	-3.4	
AT3G56940	12	CRD1	Mg-Protoporphyrin IX monomethyl ester (oxidative) cyclase	1.14.13.81		-2.4	-1.5	-2.9	
AT5G54190	13	PORA	Protochlorophyllide reductase A	1.3.1.33	3.4	-3.1	-2.5	-36.9	
AT4G27440		PORB	Protochlorophyllide reductase B			-4.2	-1.7	-4.0	
AT1G03630		PORC	Protochlorophyllide reductase C			-2.9	-2.1	-3.8	
AT5G18660	14	DVR	3,8-Divinylprotochlorophyllide *a* 8-vinyl reductase	1.3.1.75		-3.3	-1.8	-3.0	
Phytol tail synthesis									
AT1G74470		CHLP	Geranylgeranyl diphosphate reductase (phytyl diphosphate synthesis)	1.3.1.83		-1.9		-3.1	
Chlorophyll a/b synthesis									
AT3G51820	15	CHLG	Chlorophyll a synthetase	2.5.1.62		-2.4	-1.5	-2.6	
AT1G44446	16	CAO	Chlorophyllide *a* oxygenase	1.13.12.14		-1.8	-1.8	-2.7	
AT4G13250	17	NYC1	Chlorophyll b reductase	1.1.1.294					
AT5G04900		NOL	Chlorophyll(ide) b reductase NOL, chloroplastic			-2.4	-1.7	-2.1	
AT1G04620	18	HCAR	7-Hydroxymethyl chlorophyll a reductase	1.17.7.2		-2.8	-1.6	-2.4	
**Chlorophyll Catabolism**									
Fruit Ripening, Pathogen/Wounding Pathway									
AT1G19670	20	CHL1	Chlorophyllase 1	3.1.1.14	1.8	-1.6			
AT5G43860		CHL2	Chlorophyllase 2		3.4	2.8			
AT4G22920	21	SGR1	Magnesium dechelatase	4.99.1.10		2.7		1.8	
AT3G44880	22	PAO	Pheophorbide a oxgenase (PAO)				-1.6	1.7	
AT4G16690	23	PPD	Pheophorbidase	3.1.1.82	1.7				
AT4G37000	24	RCCR	Red chlorophyll catabolite reductase	1.3.1.80			-1.6		
Senescence Pathway									
AT4G22920	21	SGR1	Magnesium dechelatase	4.99.1.10		2.7		1.8	
AT5G13800	25	PPH	Pheophytinase (PPH)	3.1.1.-					
**Regulation of chlorophyll biosynthetic process (GO:0010380)**									
AT3G22840		ELIP1	Early light-induced protein 1		1.5	-7.5	-3.1		
AT4G14690		ELIP2	Early light-induced protein 2			-5.7	-2.1		
AT5G56860		GATA21	GATA transcription factor 21		-1.6	-1.9	-1.7	-2.8	
AT4G26150		GATA22	GATA transcription factor 22		-3.0	-3.8	-2.1		
AT5G44190		GLK2	Transcription activator GLK2		-2.0	-2.4	-1.9	-2.9	
AT2G20180		PIF1	Transcription factor PIF1			-1.6	-1.5		

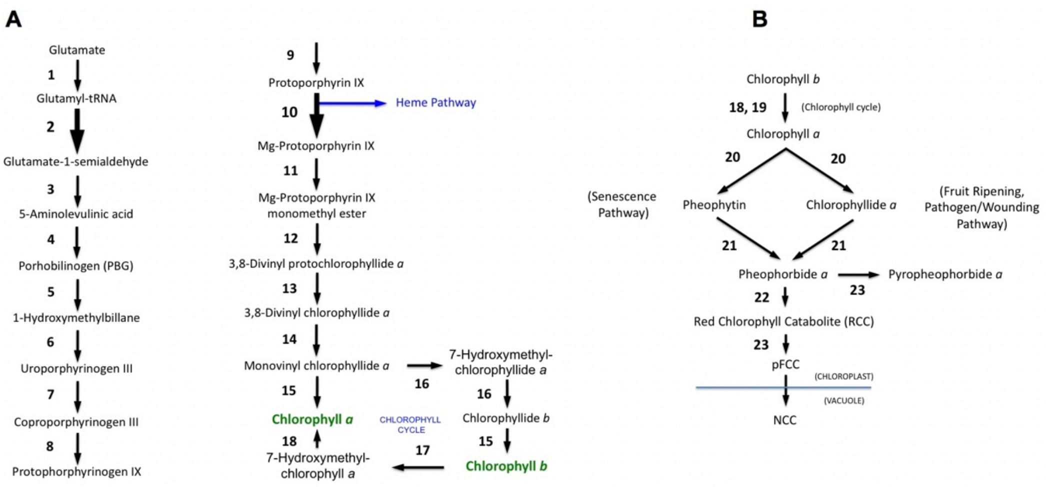

Supplemental Figure 4. Effects of pyrimidine limitation on expression of genes encoding enzymes in the isoprenoid synthesis (A) and carotenoid synthesis (B) pathways. Fold-change (FC) values for DE genes are presented as a heatmap at day 5, 7 and 9 time points (C = Control, P = PALA treatment, PU = PALA + Urd treatment). Expression values for untreated controls were the reference at each time point. Enzyme abbreviations are listed in the table. Isoprenoid pathway intermediates are listed in **Supplemental Table 3**.

AGI	Symbol	Annotation	Fold-Change Expression				
			Day5 CvP	Day7 CvP	Day7 CvPU	Day9 CvP	Day9 CvPU
**MEP Pathway**							
AT4G15560	DXS2	1-Deoxy-D-xylulose-5-phosphate synthase 2		-1.8	-1.7	-3.0	
AT5G62790	DXR	1-Deoxy-D-xylulose-5-phosphate reductoisomerase 1		-2.3	-1.7	-1.8	
AT2G02500	MCT	2-C-Methyl-D-erythritol 4-phosphate cytidylyltransferase	-1.5	-3.8	-1.6	-3.4	
AT2G26930	CMK	4-Diphosphocytidyl-2-C-methyl-D-erythritol kinase		-2.5	-1.5	-1.8	
AT1G63970	MDS	2-C-Methyl-D-erythritol 2,4-cyclodiphosphate synthase		-1.8	-1.6		
AT5G60600	HDS1	4-Hydroxy-3-methylbut-2-en-1-yl diphosphate synthase 1				-1.7	
AT4G34350	HDR	4-Hydroxy-3-methylbut-2-enyl diphosphate reductase				-1.6	
**Prenyl Transferases**							
AT5G47770	FPS2	Farnesyl pyrophosphate synthase 2		-1.5			
AT4G36810	GGPS1	Geranylgeranyl pyrophosphate synthase 1		-1.8	-1.5		
**Carotenoid biosynthesis**							
Lycopene Synthesis							
AT5G17230	PSY1	Phytoene synthase 1		-2.3	-2.2	-3.4	
AT4G14210	PDS	15-cis-phytoene desaturase		-1.6	-1.5	-1.9	
AT1G10830	Z-ISO	15-cis-zeta-carotene isomerase		-2.1		-2.0	
AT3G04870	ZDS1	Zeta-carotene desaturase 1		-2.0	-1.7	-1.8	
AT1G06820	CRTISO	Prolycopene isomerase		-1.6		-1.7	
α-Carotene Pathway							
AT5G57030	LUT2	LUTEIN DEFICIENT 2		-2.2	-1.9	-2.9	
AT1G31800	LUT5	LUTEIN DEFICIENT 5		-2.4	-1.7	-2.5	
AT3G53130	LUT1	LUTEIN DEFICIENT 1		-2.5	-1.7	-2.9	
β-Carotene Pathway							
AT3G10230	LCYB	Lycopene beta cyclase 1				-1.7	
AT4G25700	BCH1	Beta-carotene 3-hydroxylase 1		-2.5	-1.7	-2.9	
AT5G67030	ZEP	Zeaxanthin epoxidase			-1.7	-1.7	

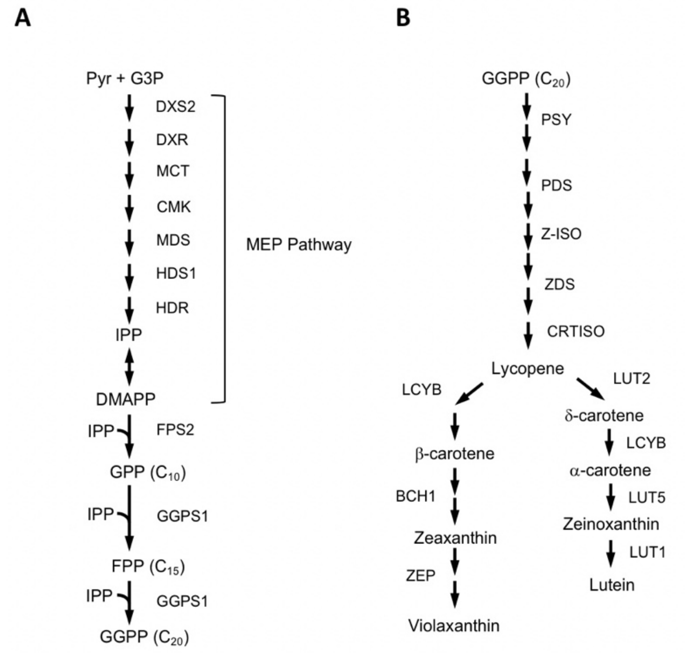

Supplemental Table 1. Primer pairs used for qRT-PCR verification of gene expression for 35 target genes, five spike controls and four invariant controls, with predicted product sizes for each.

AGI Locus	Sequence (5' to 3')	Product size (bp)	AGI Locus	Sequence (5' to 3')	Product size (bp)
AT1G10540_L	CCTCCAAAGGACAAGACGAC	120	AT3G46940_L	CTGGTTTGGCTTGGAAACAT	127
AT1G10540_R	CTTGCTGAGATTGAGAGGCA		AT3G46940_R	CGATCACCGAACTTAACCTCA	
AT1G16350_L	TCTCGGTGCTTCTTCTTTGC	100	AT3G53900-L	GCTGCACCTCCTGCACTATC	127
AT1G16350_R	GATTCCACCTTCTATCTGTGCTG		AT3G53900-R	GGTCTCCAGCGTCTCCAAG	
AT1G28230_L	GTGTTCTGCTTCCGGTGACT	146	AT3G54470_L	GTGGTGATGCACTTGGTCAG	102
AT1G28230_R	AACAACTTTCTTGCCGGATTT		AT3G54470_R	TTTCCGCCTTTATGATTCCC	
AT1G30820_L	AATCCAGCTTCTGCTCATCAA	126	AT3G57560_L	ATGTGGCTGGAATCTTGGAG	118
AT1G30820_R	GCCATTGCAGTAGCCGTTAT		AT3G57560_R	TTAGGAATCATCCCACCAGC	
AT1G52890_L	TACCTGAAGACGGAGGAGGA	124	AT4G09320_L	CCGGAAGAAAGATCATTGGA	148
AT1G52890_R	TAAACCCAAACCCACCAACT		AT4G09320_R	GGGAACCACAAAGCAATCTC	
AT1G63660-L	GTGGATCCCGAGACAAAGAG	148	AT4G23010_L	GGATCAGGCTTGCTGTTGA	125
AT1G63660-R	GGCACGACTCAATCACATCA		AT4G23010_R	TCTCTATCACCATCGCCTCC	
AT1G70330_L	ATTGGGACTTACCAACGGGT	136	AT4G34890_L	GGAATCCGAATACAAAGGCA	132
AT1G70330_R	GACAGATCCAGAGGCCAAAC		AT4G34890_R	AGTCTGTGAGACCCACCTCG	
AT1G75330_L	AGGATTCCAGGTGGATGAAG	105	AT4G34900_L	GCCGTTCCAACTGAATGTTT	145
AT1G75330_R	ACGACTCCATTGGTCACCTC		AT4G34900_R	GCTTCTAGCCGCTTTGATTG	
AT2G02990_L	TGGTTTCACTCCTTGGGTTG	103	AT5G11160_L	CTACTGGCGGCACTCTTTCT	123
AT2G02990_R	TTCGATTAAACCGGAACCAG		AT5G11160_R	GCTTCCCTTTGAGCTTGTGT	
AT2G03530_L	ATGGTCGTTACTGGGCCTTT	114	AT5G35170-L	TGATGCAAACAGGCCAAA	132
AT2G03530_R	GAAGAGCCTGAACGGAGTCA		AT5G35170-R	CTCGTCGGTATTCCTCTCCA	
AT2G16570_L	ACTATCCCGTGAAGCCAACA	109	AT5G63310_L	CAAGATGTTTCAGTGCCCAA	124
AT2G16570_R	TATCGAACCCAACCTGCTTC		AT5G63310_R	TTCCCAAGCCATACACACAA	
AT2G19570_L	GGATTGTGACGGGAAAGTGT	136	AT5G64370_L	CGGCAATACAAGGACAAGTG	112
AT2G19570_R	TCCGACGATCCTCTCGTATC		AT5G64370_R	GGTCAGAGACGACTTGAGGC	
AT2G35040_L	AGCAGAACCTGGAGGCAGTA	101			
AT2G35040_R	AATGGCGGAAATGTCTAACG		Spike Controls		
AT2G37500_L	TCAACCTCTTCCGTTTGACAG	103			
AT2G37500_R	TGCACCATCACCTACGGATA		thrC_L	atccggaaacaatagcgaca	134
AT2G41880_L	CTTCCCATTGAGTACGCAGTAT	115	thrC_R	cagctgatatgcgtgaagga	
AT2G41880_R	GTGCTCCTCCATTAAGCGAA		trpF_L	agagacgatgtcacggattg	126
AT3G07800_L	CCTGTTTGTCGCAAGCATTA	105	trpF_R	tgaagcgccttccatatttc	
AT3G07800_R	AGCAGCAACACAGGATTCAG		lysA_L	tcatgccactgaatacgttga	131
AT3G08860_L	CGCAGGACACGCAGTACTTA	128	lysA_R	aaagagttgtgcctgcgtct	
AT3G08860_R	TCACGTCTCCAATGAGTTCG		dapB_L	aaattggcagaacgaacacc	101
AT3G17810_L	GGCATTCGCTGCAGTACTTT	116	dapB_R	gcatctgactcaacaggcatc	
AT3G17810_R	CCCGGTCCAATCTTTATCAG		pheB_L	ttcagtagcagatgccgttc	141
AT3G18680-L	CAAATGTTTGATGGCGTGTTC	103	pheB_R	gcacctgacctatcctccaa	
AT3G18680-R	ACAGAGAGGTCCTTTGAGGTG				
AT3G20330_L	AGATTGGATGAAATCACCGC	118	Invariant Controls		
AT3G20330_R	ACCAACCGACAAGCAGTAGC				
AT3G27060_L	CGGGAAGGTGTACGGTGTTA	122	AT4G26410_L	CCTGGAAGGGATGCTATCAA	105
AT3G27060_R	CGCTAGACATAACGGAAGCC		AT4G26410_R	GTCCGACATACCCATGATCC	
AT3G27190_L	CTTATCTCAGCGCCAGAAGG	132	AT5G15710_L	GCACTTCTGAGACTTTCGGC	148
AT3G27190_R	ATCACCAAACTCACCCAAGC		AT5G15710_R	ATGACTGAAGAGCACAACCG	
AT3G27690_L	TTCGACCCGTTGAACTTAGC	126	AT4G27960_L	TAACCATCCATTTCCCTCCA	147
AT3G27690_R	ACCGGTAACTATGGCTTGGA		AT4G27960_R	TGGAAATTGTGAGAGCAGGA	
AT3G27740_L	CGGTTGATCCAGCTTCACTT	127	AT5G46630_L	GTGCCAATGTTCACAGCATC	118
AT3G27740_R	GAGGCTTCAGGGTGGTACTG		AT5G46630_R	TGATCTCGTAAGATCCCGCT	

Supplemental Table 2. Expression profiling for genes of nucleotide metabolism and arginine synthesis. Fold-change (FC) values for DE genes are presented as a heatmap at day 5, 7 and 9 time points (C = Control, P = PALA treatment, PU = PALA + Urd treatment). Expression values for untreated controls were the reference at each time point. Relative expression for selected genes (gray) were verified by qRT-PCR analyses (see **Supplemental Figure 1**).

AGI	Symbol	Annotation	Fold-Change Expression				
			Day5 CvP	Day7 CvP	Day7 CvP+U	Day9 CvP	Day9 CvP+U
***De Novo* Synthesis**							
Pyrimidines							
AT3G20330	ATC	Aspartate carbamoyltransferase; chloroplast	-1.7				
AT1G29900	CPSB	Carbamoylphosphate synthase (large subunit); chloroplast					
AT3G27740	CPSA	Carbamoylphosphate synthase (small subunit); chloroplast	-1.6				
AT4G22930	DHO	Dihydroorotatase; cytosol					
AT5G23300	DHODH	Dihydroorotate dehydrogenase; mitochondrion	-1.8				
AT3G54470	UMPS	UMP Synthase (bifunctional); cytosol		-1.6			
Purines							
AT1G36280	ASL	Adenylsuccinate lyase; chloroplast	-1.7				
AT4G18440	ASL	Adenylsuccinate lyase; chloroplast		-1.5		-1.7	
AT3G57610	ASS	Adenylsuccinate synthetase; chloroplast		-1.6			
AT2G35040	ATIC	AICAR transformylase/IMP cyclohydrolase (bifunctional); chloroplast	-1.6	-1.5		-1.5	
AT2G05140	AIRC	AIR carboxylase; chloroplast					
AT3G55010	AIRS	AIR synthase; chloroplast	-2.1	-1.8			
AT2G37690	AIRC	AIR carboxylase; chloroplast	-2.3				
AT2G38280	AMPD	AMP deaminase; chloroplast					
AT1G74260	FGAMS	FGAM synthase; chloroplast				-1.7	
AT2G16570	ASE1	Glutamine amidophosphoribosyltransferase 1, ATase 1; chloroplast		1.9	2.7		
AT4G34740	ASE2	Glutamine amidophosphoribosyltransferase 2, ATase 2; chloroplast					
AT4G38880	ASE3	Glutamine amidophosphoribosyltransferase 3, ATase 3					
AT1G09830	GARS	Glycinamide ribonucleotide (GAR) synthetase, GARS; chloroplast					
AT1G31220	GART	Glycinamide ribonucleotide (GAR) transformylase; chloroplast					
AT1G63660	GMPS	GMP synthase; chloroplast					
AT1G79470	IMPDH1	IMP dehydrogenase 1; chloroplast					
AT1G16350	IMPDH2	IMP dehydrogenase 2; chloroplast					
AT3G21110	SAICARS	SAICAR synthetase; chloroplast	-1.6	-1.9			
**Synthesis of NTP and dNTP**							
Pyrimidines							
AT1G30820	CTPS1	CTP synthase 1; cytosol	-3.1	-3.8		-2.0	
AT3G12670	CTPS2	CTP synthase 2; cytosol	-1.5				
AT4G02120	CTPS3	CTP synthase 3; cytosol					
AT4G20320	CTPS4	CTP synthase 4; cytosol		-2.5		-2.7	
AT2G34890	CTPS5	CTP synthase 5; cytosol					
AT3G46940	DUT	Deoxyuridine 5'-triphosphate nucleotidohydrolase; cytosol		-1.7			
AT2G21550	DHFR-TS3	Dihydrofolate reductase-thymidylate synthase 3; bifunctional; cytosol		2.9		3.4	
AT2G16370	DHFR-TS1	Dihydrofolate reductase-thymidylate synthase 1; bifunctional; cytosol					
AT4G34570	DHFR-TS2	Dihydrofolate reductase-thymidylate synthase 2; bifunctional; cytosol					
AT5G59440	TMK	Thymidylate kinase; cytosol, mitochondrion					
AT3G60180	UMK1	UMP/CMP Kinase 1; cytosol, mitochondrion		1.7		1.8	
AT4G25280	UMK2	UMP/CMP Kinase 2; cytosol, mitochondrion					
AT5G26667	UMK3	UMP/CMP Kinase 3; cytosol, mitochondrion					
AT3G60961	UMK4	UMP/CMP kinase 4; cytosol, mitochondrion				3.8	
AT3G18680	PUMPKIN	UMP/CMP Kinase 5; plastid UMP kinase, PUMPKIN		-3.1	-1.6	-1.9	
AT3G10030	UMK6	UMP/CMP Kinase 6; cytosol, mitochondrion					
Purines							
AT2G37250	AMK1	AMP kinase 1; chloroplast					
AT5G47840	AMK2	AMP kinase 2; chloroplast				-1.7	
AT5G50370	AMK3	AMP kinase 3; cytosol					
AT5G63400	AMK4	AMP kinase 4; cytosol					
AT5G35170	AMK5	AMP kinase 5; chloroplast		-1.6		-3.3	
AT2G39270	AMK6	AMP kinase 6; chloroplast		2.0		2.4	
AT3G01820	AMK7	AMP kinase 7; mitochondrial					
AT5G60340	AAK6	AMP kinase 6; nucleus					
AT2G41880	GMK1	GMP kinase 1; cytosol				-1.8	
AT3G57550	GMK2	GMP kinase 2; cytosol		1.8			
AT3G06200	GMK3	GMP kinase 3; chloroplast, mitochondrion					
Pyrimidines and Purines							
AT2G21790	RNR1	NDP reductase (large subunit); cytosol					
AT3G23580	RNR2A	NDP reductase (small subunit); cytosol					
AT5G40942	RNR2B	NDP reductase (small subunit); cytosol					
AT3G27060	TSO2	NDP reductase (small subunit); cytosol					
AT4G09320	NDPK1	Nucleoside diphosphate kinase 1; cytosol	-1.7				
AT5G63310	NDPK2	Nucleoside diphosphate kinase 2; chloroplast		-2.3		-2.2	
AT4G11010	NDPK3	Nucleoside diphosphate kinase 3; chloroplast, mitochondrion					
AT4G23900	NDPK4	Nucleoside diphosphate kinase 4; chloroplast, mitochondrion					
AT1G17410	NDPK5	Nucleoside diphosphate kinase 5; cytosol, chloroplast					
**Salvaging (Intracellular)**							
Pyrimidines							
AT2G19570	CDA1	Cytidine deaminase 1; cytosol					
AT4G29620	CDA2	Cytidine deaminase 2; cytosol (pseudogene)					
AT4G29630	CDA3	Cytidine deaminase 3; cytosol (pseudogene)					
AT4G29650	CDA4	Cytidine deaminase 4; cytosol (pseudogene)					
AT4G29640	CDA5	Cytidine deaminase 5; cytosol (pseudogene)					
AT4G29610	CDA6	Cytidine deaminase 6; cytosol (pseudogene)					
AT4G29600	CDA7	Cytidine deaminase 7; cytosol (pseudogene)					
AT4G29570	CDA8	Cytidine deaminase 8; cytosol (pseudogene)		2.9		9.8	
AT4G29580	CDA9	Cytidine deaminase 9; cytosol (pseudogene)					
AT3G07800	TK1a	Thymidine kinase 1a; cytosol, chloroplast, mitochondrion	1.9	1.8		1.7	1.7
AT5G23070	TK1b	Thymidine kinase 1b; cytosol, chloroplast, mitochondrion	-1.8	-2.8	-1.5		
AT1G06730	PNK1	PLASTID NUCLEOSIDE KINASE 1		-1.6			
AT3G53900	UPRT	Uracil phosphoribosyltransferase; UMP phosphorylase, UPP; chloroplast		-3.0	-1.5	-2.1	
AT5G40870	UCK1	Uridine cytidine kinase 1; cytosol					
AT3G27190	UCK2	Uridine cytidine kinase 2; cytosol	1.7	2.1	1.8	2.3	
AT1G55810	UCK3	Uridine cytidine kinase 3; cytosol					
AT4G26510	UCK4	Uridine cytidine kinase 4; cytosol					
AT3G27440	UCK5	Uridine cytidine kinase 5; cytosol					
Purines							
AT1G27450	APT1	Adenine phosphoribosyltransferase 1; chloroplast					
AT1G80050	APT2	Adenine phosphoribosyltransferase 2; PM, Golgi			1.6	1.9	
AT4G22570	APT3	Adenine phosphoribosyltransferase 3; cytosol		-2.0			
AT4G12440	APT4	Adenine phosphoribosyltransferase 4; cytosol					
AT5G11160	APT5	Adenine phosphoribosyltransferase 5; cytosol		-1.5			
AT3G09820	ADK1	Adenosine kinase 1; cytosol					
AT5G03300	ADK2	Adenosine kinase 2; cytosol					
AT1G71750	HGPRT	Hypoxanthine-guanine phosphoribosyltransferase; cytosol					
Pyrimidines and Purines							
AT1G72040	dNK	Deoxynucleoside kinase; cytosol, mitochondria	-1.5	-2.0			
AT2G39780	RNS2	Ribonuclease 2; vacuole, ER	1.6				
AT2G35390	PRS1	PRPP Synthase 1; chloroplast				-2.0	
AT1G10700	PRS3	PRPP Synthase 3; chloroplast	1.6	1.5			

AGI	Symbol	Annotation	Fold-Change Expression				
			Day5 CvP	Day7 CvP	Day7 CvP+U	Day9 CvP	Day9 CvP+U
**Catabolism**							
Pyrimidines							
AT3G08860	PYD4	β-alanine-pyruvate aminotransferase 3; cytosol	4.9	4.3		4.3	
AT5G64370	PYD3	β-ureidopropionase; cytosol	2.0	2.2		1.8	
AT5G12200	PYD2	Dihydropyrimidinase; endoplasmic reticulum	1.5	1.6			
AT3G17810	PYD1	Dihydropyrimidine dehydrogenase; chloroplast		1.5		1.9	
Purines							
AT4G20070	AAH	Allantoate amidohydrolase; endoplasmic reticulum				-1.7	
AT5G58220	ALNS	Allantoin synthase; peroxisome	1.6	1.6		1.7	
AT4G04955	ALN	Allantoinase; endoplasmic reticulum					
AT5G28050	GSDA	Guanosine deaminase; cytosol	1.5				
AT4G17050	UGAH	Ureidoglycine aminohydrolase; endoplasmic reticulum					
AT5G46300	UAH	Ureidoglycolate aminohydrolase; endoplasmic reticulum					
AT2G26230	UOX	Uricase; peroxisome		1.5			
AT4G34890	XDH1	Xanthine dehydrogenase 1; cytosol	1.7	1.8			
AT4G34900	XDH2	Xanthine dehydrogenase 2; cytosol (inactive)		-2.1		-4.0	
Pyrimidines and Purines							
AT2G36310	NSH1	Nucleoside hydrolase 1; cytosol				1.5	
AT1G05620	NSH2	Nucleoside hydrolase 2; cytosol			1.6	1.9	
**Salvaging (Extracellular)**							
Nucleotidases, Nucleosidases							
AT1G14250	APY5	Apyrase 5; ER, PM	11.1	2.6		3.9	
AT2G02970	APY6	Apyrase 6; ER, PM	1.6	1.5			
AT4G29690	NPP3	Ecto-nucleotide pyrophosphatase / alkaline phosphodiesterase 3; cell wall, vacuole	2.9		2.6		
AT5G18860	NSH3	Nucleoside hydrolase 3 (purine-specific); cell wall		2.3	2.1		
Non-Specific Phosphatases							
AT1G13750	PAP1	Purple acid phosphatase 1 (inactive); cell wall	3.9	6.6	2.0	3.1	
AT1G25230	PAP4	Purple acid phosphatase 4; cell wall	2.4	2.4	2.0		
AT2G01890	PAP8	Purple acid phosphatase 8; cell wall	1.7	1.8		1.8	
AT2G16430	PAP10	Purple acid phosphatase 10; cell wall	1.5	2.2		1.7	
AT3G07130	PAP15	Purple acid phosphatase 15; cell wall				2.0	
AT3G20500	PAP18	Purple acid phosphatase 18; cell wall		1.6		2.1	
AT5G50400	PAP27	Purple acid phosphatase 27 (inactive); cell wall		1.7		1.7	
Nucleases							
AT2G02990	RNS1	Ribonuclease 1; cell wall (secreted)	12.3	8.3		11.8	
AT1G14210	RNST2	Ribonuclease T2, cell wall (secreted)				2.2	
Transporters (PM-localized) for Cellular Uptake of Nucleobases, Nucleosides							
AT4G05120	ENT3	Equilibrative nucleoside transporter 3	1.8				
AT1G60030	NAT7	Nucleobase:cation symporter 1 family (NCS1)/Nucleobase-ascorbate transporter 7		3.2		2.4	
AT1G10540	NAT8	Nucleobase:cation symporter 1 family (NCS1)/Nucleobase-ascorbate transporter 8		3.2		5.1	
AT3G10960	AtAzg1	Nucleobase:cation symporter 2 family (NCS2/AZGA-like protein family); AtAzg1				1.7	
AT5G50300	AtAzg2	Nucleobase:cation symporter 2 family (NCS2/AZGA-like protein family); AtAzg2			2.6		
AT2G03530	UPS2	Ureide permease 2		2.0			
AT1G26440	UPS5	Ureide permease 5		2.3	1.6		
AT1G28230	PUP1	Purine permease 1	8.0	8.5		4.2	
AT4G18210	PUP10	Purine permease 10		4.1		3.3	
AT5G41160	PUP12	Purine permease 12		4.1			
AT1G19770	PUP14	Purine permease 14	1.8		1.6	-2.7	
AT1G57990	PUP18	Purine permease 18	2.6	3.0	1.8	-2.3	
Other Nucleobase, Nucleoside Transporters							
AT1G70330	ENT1	Equilibrative nucleoside transporter 1; tonoplast	1.6				
AT5G03555	PLUTO	Nucleobase:cation symporter 1 family (NCS1), PLUTO			-1.5		
AT2G34190	NAT2	Nucleobase:cation symporter 1 family (NCS1)/Nucleobase-ascorbate transporter 2	-1.7	-2.9			
AT2G33750	PUP2	Purine permease 2		-1.6			
AT1G57980	PUP23	Purine permease 23				-2.1	
AT1G28220	PUP3	Purine permease 3		-1.5			
AT1G30840	PUP4	Purine permease 4			1.6		
AT2G24220	PUP5	Purine permease 5		-1.6			
AT4G18220	PUP9	Purine permease 9		-2.2	-2.0		
Nucleotide Transporters							
AT1G80300	AtNTT1	ATP:ADP transporter 1; chloroplastic		1.6		1.5	
AT1G15500	AtNTT2	ATP:ADP transporter 2; chloroplastic		-1.5			
AT4G32400	BT1	BRITTLE1, adenylate uniport carrier					
**Arginine Synthesis**							
AT2G22910	NAGS1	*N*-acetylglutamate synthase 1					
AT4G37670	NAGS2	*N*-acetylglutamate synthase 2					
AT3G57560	NAGK	*N*-acetylglutamate kinase					
AT2G19940	NAGPR	*N*-acetylglutamate-5-P reductase					
AT1G80600	NAOAT	*N2*-acetylornithine aminotransferase					
AT2G37500	NAOGAT	*N2*-acetylornithine:glutamate acetyltransferase					
AT4G17830	NAOD	*N2*-acetylornithine deacetylase				1.6	
AT1G75330	OTC	Ornithine transcarbamylase		-1.6			
AT4G24830	AS	Argininosuccinate synthase	-1.6	-1.5			
AT5G10920	AL	Argininosuccinate lyase	-1.6	-1.7			
AT4G01900	PII	PII, N-sensing regulatory protein		-2.1	-1.6		
Gene expression verified by qRT-PCR							

Supplemental Table 3. Abbreviations used for pathway intermediates or enzymes not annotated in previous tables or pathway diagrams.

Ade	adenine	Guo	guanosine
Ado	adenosine	HIU	5-hydroxyisourate
ADP	adenosine-5'-diphosphate	IMP	inosine-5'-monophosphate
AICAR	5-aminoimidazole-4-carboxamide	IPP	isopentenyl diphosphate
AIR	5-aminoimidazole ribonucleotide	NAG	*N*-acetylgluatamate
AL	argininosuccinate lyase	NAGK	*N*-acetylglutamate kinase
AMP	adenosine-5'-monophosphate	NAGP	*N*-acetylglutamate-5-phosphate
Arg	L-arginine	NAGPR	*N*-acetylglutamate-5-P reductase
AS	argininosuccinate synthase	NAGS	*N*-acetylglutamate synthase
Asp	L-aspartate	NAGSA	*N*-acetylgluatamate-5-semialdehyde
ATP	adenosine-5'-triphosphate	NAO	*N2*-acetylornithine
CAIR	caroxyaminoimidazole	NAOAT	*N2*-acetylornithine aminotransferase
CarAsp	carbamoyl aspartate	NAOD	*N2*-acetylornithine deacetylase
CDP	cytidine-5'-diphosphate	NAOGAT	*N2*-acetylornithine:glutamate acetyltransferase
CP	carbamoyl phosphate	OHCU	2-oxo-4-hydroxy-4-carboxy-5-ureidoimidazoline
CTP	cytidine-5'-triphosphate	Orn	L-ornithine
Cyd	cytidine	OTC	ornithine transcarbamylase
dCyd	deoxycytidine	PALA	*N*-(phosphonacetyl)-L-aspartate
DMAP	dimethylallyl diphosphate	PII	PII nitrogen regulatory protein
FAICAR	5-formaminoimidazole-4-carboxamide ribonucleotide	PRA	phosphoribosylamine
FGAM	formylglycinamidine ribonucleotide	PRPP	5-phosphoribosyl-1-pyrophosphate
FGAR	formylglycinamide ribonucleotide	Pyr	pyruvate
FPP	farnesyl diphosphate	SAICAR	5-aminoimidazole-4-(N-succinylcarboxamide) ribonucletide
G3P	glyceraldehyde-3-phosphate	SAMP	adenylosuccinate
GAR	glycinamide ribonucleotide	Thd	thymidine
GDP	guanosine-5'-diphosphate	UDP	uridine-5'-diphosphate
GGPP	geranyl geranyl diphosphate	UMP	uridine-5'-monophosphate
Gln	L-glutamine	Ura	uracil
Glu	L-glutamate	Urd	uridine
GMP	guanosine-5'-monophosphate	USP	UDP-sugar pyrophosphorylase
GPP	geranyl diphosphate	UTP	uridine-5'-triphosphate
GTP	guanosine-5'-triphosphate	XMP	xanthosine-5'-monophosphate
Gua	guanine		

Supplemental Table 4. Transcription factor enrichment analysis for genes encoding enzymes and transporters of nucleotide metabolism and arginine synthesis in Arabidopsis. TF with over-represented targets in one or more groups of coexpressed genes are indicated in the top row. Target genes for each TF are indicated. Annotation and expression profiles for these TF are found in **Table S5**. Bold TF symbols indicate TF whose expression was highly-responsive (induction or repression) to PALA and was reversed by Urd. A few TF were responsive to Urd but not PALA, or responsive to PALA, without Urd reversal. Some co-expressed genes were not DE in the present study but are included since they are functionally-related and share TF targets with other highly coexpressed genes in the group.

TF With Enriched Targets (column headers):

Group	TF
Ethylene-Responsive TF	ABR1, CRF2, ERF008, **ERF011**, ERF37, ERF38, ERF073, ERF104, ERF105, **ERF115**, **ERF1**, ERF2, ERF3, ERF4, ERF5, ERF6, ERF9, **ERF11**, ERF12, ERF13, ERF15, LEP, RAP2-3, RAP2-4, **RAP2-6**, RAP2-9, RAP2-11, RAP2-12
Miscellaneous TF	ARF3, ASIL2, ASR3, ATHB-5, ATHB-6, ATHB-13, AZF1, bHLH10, bHLH74, bHLH80, bHLH130, BZIP11, BZIP44, CDF5, DOF1.6, DOF2.2, DOF3.4, DOF5.6, DOF5.8, DREB1B, DREB1C, DREB2C, DREB2D, DREB2F, E2Fd DEL2, FUS3, GATA-1, GATA-12, GEBP, HHO5, HSFB2A, IDD4, IDD11, LBD6, LBD13, MUG13.5, MYB10, MYB13, **MYB15**, MYB17, MYB23, MYB44, **MYB51**, MYB93, **MYB111**, **NAC002**, **NAC047**, NAC096, NLP4, PHL1, PHL12, RAV1, REM16, RVE4, SEP3, SOC1, SPL11, STY1, TCP7, TCP15, TCP19, TCP20, TCP21, TCP22
WRKY TF	WRKY6, **WRKY8**, WRKY11, WRKY15, WRKY17, WRKY21, WRKY22, WRKY25, WRKY26, **WRKY28**, WRKY29, WRKY31, WRKY33, **WRKY40**, WRKY42, WRKY43, WRKY45, **WRKY46**, WRKY47, WRKY50, WRKY59, WRKY62, WRKY71, **WRKY75**

Marked targets (TF columns with a filled cell for each gene):

AGI	Symbol	Co-Expressed Genes	Ethylene-Responsive TF	Miscellaneous TF	WRKY TF
***De Novo* Synthesis**					
AT3G55010	AIRS	AIR synthase	**RAP2-6**	ASIL2, IDD11, NLP4, SOC1	
AT3G57610	ASS	Adenylsuccinate synthetase		bHLH80, bHLH130, IDD4, SOC1	
AT3G20330	ATC	Aspartate transcarbamylase; chloroplast	ABR1	ASIL2, E2Fd DEL2, SOC1	
AT2G35040	ATIC	AICARFT/IMPCHase, AICAR transformylase	ABR1, CRF2, ERF008, **ERF011**, ERF37, ERF38, ERF073, ERF104, ERF105, **ERF115**, **ERF1**, ERF2, ERF3, ERF4, ERF5, ERF6, ERF9, **ERF11**, ERF13, ERF15, LEP, **RAP2-6**, RAP2-9, RAP2-11, RAP2-12	ATHB-5, bHLH10, CDF5, DOF5.6, DREB2C, DREB2D, GATA-1, LBD6, MYB44, SOC1	
AT3G27740	CPSA	Carbamoylphosphate synthase (small subunit)		HHO5, MYB10, MYB13, **MYB15**, MYB17, **MYB51**, MYB93, **MYB111**	WRKY6, WRKY11, WRKY15, WRKY17, WRKY21, WRKY22, WRKY25, WRKY26, **WRKY28**, WRKY29, WRKY33, **WRKY40**, WRKY42, WRKY43, WRKY45, **WRKY46**, WRKY47, WRKY50, WRKY59, WRKY71, **WRKY75**
AT1G29900	CPSB	Carbamoylphosphate synthase (large subunit)		NLP4	WRKY6, **WRKY8**, WRKY11, WRKY15, WRKY17, WRKY21, WRKY22, WRKY25, WRKY26, **WRKY28**, WRKY29, WRKY31, WRKY33, **WRKY40**, WRKY42, WRKY43, WRKY45, **WRKY46**, WRKY47, WRKY50, WRKY59, WRKY71, **WRKY75**
AT1G74260	FGAMS	FGAM synthase	**ERF1**, ERF13	MYB23	
AT1G09830	GARS	GAR synthetase		TCP20	
AT1G63660	GMPS	GMP synthase		NLP4	WRKY17, **WRKY28**, WRKY45, WRKY50, WRKY59
AT3G21110	SAICARS	SAICAR synthetase		DOF1.6, DOF5.6, **MYB15**	
AT3G54470	UMPS	UMP Synthase		DOF2.2, DOF3.4, IDD11, NLP4, RAV1, SEP3, SOC1	
**NTP Synthesis**					
AT5G50370	AMK3	AMP kinase 3		MYB10, MYB13, MYB93	
AT3G12670	CTPS2	CTP synthase 2	ABR1, CRF2, ERF073, **ERF1**, ERF3, ERF4, ERF13, ERF15, **RAP2-6**, RAP2-12		
AT3G46940	DUT	Deoxyuridine 5'-triphosphate nucleotidohydrolase		AZF1	
AT4G34570	DHFR-TS2	Dihydrofolate reductase-thymidylate synthase 2	ABR1, ERF073, ERF104, ERF105, **ERF115**, **ERF1**, ERF3, ERF4, ERF5, ERF6, ERF9, **ERF11**, ERF13, ERF15, LEP, **RAP2-6**, RAP2-11, RAP2-12	IDD4	
AT4G09320	NDPK1	Nucleoside diphosphate kinase 1		PHL1	
AT5G63310	NDPK2	Nucleoside diphosphate kinase 2	ABR1, CRF2, **ERF011**, ERF073, ERF104, ERF105, **ERF1**, ERF3, ERF4, ERF5, ERF6, ERF9, **ERF11**, ERF13, ERF15, LEP, **RAP2-6**, RAP2-11, RAP2-12	ASIL2, DREB2D	
AT3G27060	TSO2	NDP reductase (small subunit)		E2Fd DEL2, REM16	
**Salvaging (Intracellular)**					
AT3G09820	ADK1	Adenosine kinase 1	ERF008, **ERF011**, ERF37, ERF38, RAP2-9	AZF1, bHLH10, DREB2D, GATA-12, MYB10, MYB13, **MYB15**, MYB17, **MYB51**, **MYB111**	
AT5G03300	ADK2	Adenosine kinase 2		DOF1.6, MYB93, NLP4, PHL1	
AT1G27450	APT1	Adenine phosphoribosyltransferase 1			WRKY6, WRKY25, WRKY26, WRKY33, WRKY47
AT1G72040	dNK	Deoxynucleoside kinase		TCP20	
AT2G39780	RNS2	Ribonuclease 2		ASIL2, ASR3, DOF5.6, IDD11, LBD6, LBD13, PHL1, PHL12	
AT3G07800	TK1a	Thymidine kinase 1a		E2Fd DEL2, REM16	
AT3G53900	UPRT	Uracil phosphoribosyltransferase		MUG13.5	
AT3G27190	UCK2	Uridine cytidine kinase 2		PHL1, PHL12	
**Catabolism**					
AT5G58220	ALNS	Allantoin synthase		DOF5.6, IDD11	
AT1G05620	NSH2	Nucleoside hydrolase 2		RAV1	
AT5G12200	PYD2	Dihydropyrimidinase		DOF5.6, RAV1	
AT2G26230	UOX	Uricase		SEP3	
AT4G34890	XDH1	Xanthine dehydrogenase 1		DOF5.6, GATA-1, SEP3	
**Salvaging (Extracellular)**					
AT4G05120	ENT3	Equilibrative nucleoside transporter 3			WRKY17
AT4G29690	NPP3	Nucleotide pyrophosphatase/phosphodiesterase 3		ASIL2, DOF5.6	
AT3G07130	PAP15	Purple acid phosphatase 15	ABR1, ERF13, LEP, RAP2-12	ASIL2, DOF5.6, DREB2C, DREB2D, DREB2F, LBD6, LBD13	
**Transporters**					
AT4G32400	BT1	BRITTLE1, adenylate uniport carrier	ABR1, CRF2, ERF073, **ERF115**, **ERF1**, ERF9, LEP, RAP2-12	GATA-12, MYB10, MYB13, **MYB15**, MYB17, **MYB51**, MYB93, **MYB111**	
AT1G70330	ENT1	Equilibrative nucleoside transporter 1	LEP	ASR3, DREB2C, DREB2D, DREB2F	
AT1G28230	PUP1	Purine permease 1		**NAC047**	
AT4G18220	PUP9	Purine permease 9		BZIP11, BZIP44, FUS3, SPL11	
AT4G18210	PUP10	Purine permease 10		TCP7, TCP15, TCP19, TCP20, TCP21, TCP22	WRKY62
AT1G19770	PUP14	Purine permease 14	ERF008, ERF37, ERF105, **ERF1**, ERF2, ERF6, ERF12, RAP2-3	ARF3, bHLH74, bHLH130, DREB1B, DREB1C, MYB23, PHL1	WRKY59
AT1G57990	PUP18	Purine permease 18	CRF2, ERF105, ERF12, ERF13, RAP2-3, RAP2-4	HSFB2A, **NAC002**	
AT1G57980	PUP23	Purine permease 23		ATHB-5, ATHB-6, ATHB-13, HSFB2A, MYB44, RVE4	
AT2G03530	UPS2	Ureide permease 2	ABR1, CRF2, ERF104, **ERF1**, ERF2, ERF5, ERF6, ERF9, ERF13, ERF15, LEP, **RAP2-6**, RAP2-12	ASIL2, CDF5, DOF2.2, DOF3.4, DOF5.8, DREB2C, GEBP, HHO5, IDD11, SOC1	
**Ornithine/Arginine Synthesis**					
AT3G57560	NAGK	N-Acetylglutamate kinase	ABR1, CRF2, ERF073, ERF104, ERF105, **ERF115**, **ERF1**, ERF3, ERF4, ERF5, ERF6, ERF9, **ERF11**, ERF13, ERF15, LEP, **RAP2-6**, RAP2-11, RAP2-12	ASIL2, DREB2D	
AT2G19940	NAGPR	N-Acetylglutamate-5-P reductase		AZF1, IDD4	
AT1G80600	NAOAT	N2-Acetylornithine aminotransferase	ERF073, ERF104, ERF105, **ERF115**, ERF3, ERF4, ERF5, ERF6, **ERF11**, ERF13, **RAP2-6**, RAP2-11, RAP2-12	ASIL2, DOF1.6, PHL12	
AT2G37500	NAOGAT	N2-Acetylornithine:glutamate acetyltransferase		MUG13.5, STY1	
AT1G75330	OTC	Ornithine transcarbamylase	ABR1, CRF2, ERF073, ERF104, ERF105, **ERF115**, **ERF1**, ERF2, ERF3, ERF4, ERF5, ERF6, ERF9, **ERF11**, ERF13, ERF15, **RAP2-6**, RAP2-11, RAP2-12	ASIL2, bHLH80, bHLH130, CDF5, DOF5.6, DREB2D, LBD6, SOC1	WRKY31
AT4G24830	AS	Argininosuccinate synthetase	ERF6, ERF15, RAP2-11	AZF1, MYB23, REM16, SOC1, STY1	
AT5G10920	AL	Argininosuccinate lyase	ABR1, ERF073, **ERF1**, ERF3, ERF4, ERF5, ERF9, **ERF11**, LEP, **RAP2-6**, RAP2-12	MYB10, **MYB15**, **MYB51**, **MYB111**, SOC1	

PALA repressed, Urd reversal

PALA induced, Urd reversal

PALA non-regulated, Urd up-regulated; PALA up-/down-regulated expression, no Urd reversal

Supplemental Table 5. Annotation and expression profiles for TF with enriched targets in highly-co-expressed genes in **Supplemental Table 4**. Fold-change (FC) values for DE genes are presented as a heatmap at day 5, 7 and 9 timepoints (C = Control, P = PALA treatment, PU = PALA + Urd treatment). Expression values for untreated controls were the reference at each time point.

AGI	TF	Annotation	Expression (Fold-Change)					Response Category
			Day5 CvP	Day7 CvP	Day7 CvPU	Day9 CvP	Day9 CvPU	
AT5G01900	WRKY62	Transcription factor WRKY62	27.5	51.1	3.3			Up-regulated by PALA, Day 5 or later
AT2G46400	WRKY46	Transcription factor WRKY46	8.3	26.8	3.7			
AT1G43160	RAP2-6	Ethylene-responsive transcription factor RAP2-6	8.1	16.3	4.9	5.8		
AT1G28370	ERF11	Ethylene-responsive transcription factor ERF11	7.8	5.5	2.2			
AT4G18170	WRKY28	Transcription factor WRKY28	5.5	10.2	3.1	2.5		
AT3G01970	WRKY45	Transcription factor WRKY45	4.2	8.6		3.0		
AT4G17490	ERF6	Ethylene-responsive transcription factor ERF6	4.2	3.8	5.7	-2.7		
AT1G18570	MYB51	Transcription factor MYB51	4.1	6.6	2.3			
AT1G75490	DREB2D	Dehydration-responsive element-binding protein 2D	3.7	2.1				
AT5G47220	ERF2	Ethylene-responsive transcription factor ERF2	3.6	3.4				
AT3G23240	ERF1B	Ethylene-responsive transcription factor ERF1B	3.6	13.5	2.8			
AT3G16770	RAP2-3	Ethylene-responsive transcription factor RAP2-3	3.5	3.9				
AT3G23250	MYB15	Transcription factor MYB15	3.4	7.6	3.2			
AT5G13910	LEP	Ethylene-responsive transcription factor LEP	3.4	2.5	2.8			
AT3G15210	ERF4	Ethylene-responsive transcription factor ERF4	3.2	2.8	2.2			
AT5G47230	ERF5	Ethylene-responsive transcription factor ERF5	3.1	2.7	4.8	-3.0		
AT3G04070	NAC047	NAC domain-containing protein 47	3.1	12.1		5.1		
AT1G80840	WRKY40	Transcription factor WRKY40	3.0	10.3	6.7			
AT2G38470	WRKY33	Transcription factor WRKY33	2.8	4.5	4.0	-1.6		
AT3G50260	ERF011	Ethylene-responsive transcription factor ERF011	2.7	7.4		2.6		
AT2G44840	ERF13	Ethylene-responsive transcription factor ERF13	2.7	3.3	2.0			
AT4G06746	RAP2-9	Ethylene-responsive transcription factor RAP2-9	2.6	2.7		3.8		
AT1G72360	ERF073	Ethylene-responsive transcription factor ERF073	2.5	2.3	2.3	2.3	1.6	
AT1G13260	RAV1	AP2/ERF and B3 domain-containing transcription factor RAV1	2.4	2.5	2.1			
AT5G05090	MUG13.5	Homeodomain-like superfamily protein	2.2	2.3				
AT5G62940	DOF5.6	Dof zinc finger protein DOF5.6	2.2	2.3	1.7			
AT5G61600	ERF104	Ethylene-responsive transcription factor ERF104	2.1	2.2	2.8	-3.5		
AT4G01250	WRKY22	Transcription factor WRKY22	2.0		1.9	-4.0		
AT1G75390	BZIP44	bZIP transcription factor 44	2.0	2.1	1.9			
AT5G62020	HSFB2A	Heat stress transcription factor B-2a	2.0	2.0	1.7			
AT5G67300	MYB44	Transcription factor MYB44	1.9	3.3		2.5		
AT2G28810	DOF2.2	Dof zinc finger protein DOF2.2	1.9					
AT1G10120	bHLH074	Transcription factor bHLH74	1.8					
AT5G44210	ERF9	Ethylene-responsive transcription factor ERF9	1.7	1.6				
AT1G72010	TCP22	Transcription factor TCP22	1.7	1.6				
AT1G53910	RAP2-12	Ethylene-responsive transcription factor RAP2-12	1.6	1.7		1.7		
AT1G01720	NAC002	NAC domain-containing protein 2	1.5					
AT2G21900	WRKY59	Transcription factor WRKY59		47.5	1.9	4.8		Up-regulated by PALA, Day 7 or later
AT5G13080	WRKY75	Transcription factor WRKY75		26.8		4.0		
AT5G46350	WRKY8	Transcription factor WRKY8		15.1		6.0		
AT5G07310	ERF115	Ethylene-responsive transcription factor ERF115		10.6				
AT5G67450	AZF1	Zinc finger protein AZF1		4.6	2.6			
AT1G62300	WRKY6	Transcription factor WRKY6		4.4	2.0	3.5		
AT5G07100	WRKY26	Transcription factor WRKY26		4.4		2.3		
AT5G26170	WRKY50	Transcription factor WRKY50		4.2				
AT4G22070	WRKY31	Transcription factor WRKY31		3.9		4.9		
AT2G30250	WRKY25	Transcription factor WRKY25		3.9		1.6		
AT5G20240	PI	Floral homeotic protein PISTILLATA		3.1		2.9		
AT3G12730	PHL12	MYB family transcription factor PHL12		3.0				
AT2G31230	ERF15	Ethylene-responsive transcription factor ERF15		2.9		2.0		
AT5G64750	ABR1	Ethylene-responsive transcription factor; AP2-like ABA repressor 1		2.8				
AT4G25470	DREB1C	Dehydration-responsive element-binding protein 1C		2.7		-4.1		
AT2G40340	DREB2C	Dehydration-responsive element-binding protein 2C		2.7				
AT2G23320	WRKY15	Transcription factor WRKY15		2.6				
AT1G69570	CDF5	Cyclic dof factor 5		2.5				
AT2G42280	bHLH130	Transcription factor bHLH130		2.2		2.1		
AT4G01720	WRKY47	Transcription factor WRKY47		2.1		2.4		
AT1G28360	ERF12	Ethylene-responsive transcription factor ERF12		2.1		4.5		
AT4G23550	WRKY29	Transcription factor WRKY29		2.0		3.7		
AT5G29000	PHL1	PHR1-LIKE 1		1.9		1.6		
AT3G27010	TCP20	Transcription factor TCP20		1.9				
AT4G37180	HHO5	Transcription factor HHO5		1.7		1.5		
AT2G22430	ATHB-6	Homeobox-leucine zipper protein ATHB-6	-1.6	1.7		2.3		
AT2G45660	SOC1	SUPPRESOR OF CONSTANS 1		1.6		-2.2		
AT1G78080	RAP2-4	Ethylene-responsive transcription factor RAP2-4		1.6		1.7		
AT2G24570	WRKY17	Transcription factor WRKY17		1.6	1.5			
AT4G31550	WRKY11	Transcription factor WRKY11		1.6				
AT1G50640	ERF3	Ethylene-responsive transcription factor ERF3		1.5				
AT1G29860	WRKY71	Transcription factor WRKY71				4.8		Up-regulated by PALA, Day 9
AT3G50410	DOF3.4	Dof zinc finger protein DOF3.4				2.8		
AT1G34670	MYB93	Transcription factor MYB93				2.6		
AT3G51060	STY1	SHI RELATED SEQUENCE 1				2.3		
AT2G23340	ERF008	Ethylene-responsive transcription factor ERF008				2.1		
AT1G06180	MYB13	Transcription factor MYB13				1.9		
AT2G30590	WRKY21	Transcription factor WRKY21				1.6		
AT5G46590	NAC096	NAC domain containing protein 96	-3.6	-1.6				Down-regulated by PALA, Day 5 or later
AT1G24260	SEP3	SEPALLATA 3	-3.4	-2.6	-2.9			
AT4G34590	BZIP11	bZIP transcription factor 11	-3.2	-2.6				
AT5G66940	DOF5.8	Dof zinc finger protein DOF5.8	-2.9	-4.5				
AT5G49330	MYB111	Transcription factor MYB111	-2.6	-9.1	-2.6	-8.4		
AT1G65620	LBD6	LOB domain-containing protein 6	-2.5	-2.6				
AT5G40330	MYB23	Transcription factor MYB23	-2.4	-3.3				
AT4G23750	CRF2	Ethylene-responsive transcription factor CRF2	-2.3	-2.2				
AT3G13810	IDD11	Indeterminate-domain 11	-2.3		-1.5	1.7		
AT2G33550	ASR3	Trihelix transcription factor ASR3	-2.2			1.8		
AT1G35460	bHLH080	Transcription factor bHLH80	-1.9	-2.1				
AT1G69780	ATHB-13	Homeobox-leucine zipper protein ATHB-13	-1.9					
AT2G02080	IDD4	Indeterminate-domain 4	-1.7	-1.5	-1.7			
AT5G65310	ATHB-5	Homeobox-leucine zipper protein ATHB-5	-1.6	-1.9	-1.6			
AT3G24050	GATA-1	GATA transcription factor 1	-1.6		-1.5			
AT3G14180	ASIL2	Trihelix transcription factor ARABIDOPSIS 6B-INTERACTING PROTEIN1-LIKE2	-1.5					
AT5G23280	TCP7	Transcription factor TCP7	-1.5		-1.5			
AT3G57600	DREB2F	Dehydration-responsive element-binding protein 2F		-5.1	-1.9	-3.7		Down-regulated by PALA, Day 7 or later
AT4G33280	REM16	AP2/B3-like transcriptional factor family protein REM16		-4.6				
AT2G35700	ERF038	Ethylene-responsive transcription factor ERF038		-3.5	-2.4			
AT3G61250	MYB17	Transcription factor MYB17		-2.6				
AT2G31220	bHLH010	Transcription factor bHLH10		-2.4				
AT4G04450	WRKY42	Transcription factor WRKY42		-2.2				
AT1G47655	DOF1.6	Dof zinc finger protein DOF1.6		-2.1				
AT5G51910	TCP19	Transcription factor TCP19		-2.1				
AT2G46130	WRKY43	Transcription factor WRKY43		-1.9				
AT1G69690	TCP15	Transcription factor TCP15		-1.9		-2.4		
AT5G14960	E2Fd DEL2	E2F transcription factor-like E2FD		-1.8				
AT1G20640	NLP4	NIN-like protein 4		-1.8	-1.5			
AT1G27360	SPL11	Squamosa promoter-binding-like protein 11		-1.7		-1.9		
AT2G33860	ARF3	Auxin response factor 3		-1.6				
AT3G26790	FUS3	AP2/B3-like transcriptional factor family protein FUS3		-1.5				
AT3G12820	MYB10	Transcription factor MYB10		-1.5				
AT4G00270	GEBP	GLABROUS1 enhancer-binding protein		-1.5	-1.5			
AT1G77200	ERF037	Ethylene-responsive transcription factor ERF037		-1.5				
AT4G25490	DREB1B	Dehydration-responsive element-binding protein 1B				-3.7		Down-regulated by PALA, Day 9
AT5G02840	RVE4	REVEILLE 4				-1.8		
AT5G08330	TCP21	Transcription factor TCP21				-1.7		
AT5G51190	ERF105	Ethylene-responsive transcription factor ERF105			4.2			Urd-responsive; not regulated by PALA
AT5G25830	GATA-12	GATA transcription factor 12			2.1			
AT2G30340	LBD13	LOB domain-containing protein 13			2.6			
AT5G19790	RAP2-11	Ethylene-responsive transcription factor RAP2-11			2.4			
